# Treatment patterns and patient journey in progressive pulmonary fibrosis: a cross-sectional survey

**DOI:** 10.1186/s12931-024-02995-9

**Published:** 2024-10-09

**Authors:** Nazia Chaudhuri, Paolo Spagnolo, Claudia Valenzuela, Valeria C. Amatto, Oliver-Thomas Carter, Lauren Lee, Mark Small, Michael Kreuter

**Affiliations:** 1 Faculty of Life and Health Sciences, School of Medicine, Ulster University, Magee Campus, Londonderry, UK; 2 Respiratory Disease Unit, Department of Cardiac, Thoracic, Vascular Sciences and Public Health, University of Padova, Padova, Italy; 3 ILD Unit, Pulmonology Department, Hospital Universitario de la Princesa, Universidad Autónoma de Madrid, Madrid, Spain; 4 TA Inflammation Med, Boehringer Ingelheim International GmbH, Ingelheim am Rhein, Germany; 5 Adelphi Real World, Bollington, UK; 6 Mainz Center for Pulmonary Medicine, Department of Pneumology ZfT, Department of Pulmonary, Critical Care & Sleep Medicine, Mainz University Medical Center, Marienhaus Clinic Mainz, Mainz, Germany

**Keywords:** Pulmonary fibrosis, Progressive pulmonary fibrosis, Interstitial lung disease, Antifibrotics, Market research, Survey, Real-world data, Patient journey, Treatment patterns, Diagnosis

## Abstract

**Background:**

For patients with interstitial lung diseases (ILDs) presenting with a progressive pulmonary fibrosis (PPF) phenotype, current knowledge of disease characteristics at diagnosis, patient journey, and treatment is limited. This study aimed to describe demographics and clinical experiences of patients presenting with PPF in a European real-world setting.

**Methods:**

Data were analysed from the Adelphi Real World PPF-ILD Disease Specific Programme™, a cross-sectional survey of pulmonologists and rheumatologists in five European countries (France, Germany, Italy, Spain, United Kingdom) and internal medicine specialists (France) from April to October 2022. Physicians provided data for up to 12 consecutive patients with physician-confirmed ILD with a progressive phenotype other than idiopathic pulmonary fibrosis. Analyses were descriptive.

**Results:**

Overall, 265 physicians reported on 1,335 patients. Mean (standard deviation) age at survey date was 60.4 (11.6) years, 91.2% were white, 58.1% female, 44.0% non-smokers. Most patients (63.3%) first consulted a primary care physician. There was a mean delay of 7.8 (22.7) months between first ILD symptom and healthcare professional visit, and another 7.7 (12.8) months to ILD diagnosis. At survey date, 47.7% of patients had physician-reported moderate ILD, 42.3% had mild ILD and 10.0% had severe ILD. Disease progression was reported in the 12 months prior to the survey for 19.5% of patients; of these, progression was based on worsening symptom in 27.3% and lung function decline in 25.8%. For patients experiencing symptoms prior to ILD diagnosis (72.8%), the most common symptoms were dyspnoea on exertion (80.5%) and cough (57.8%). Overall, 17.4% of patients were misdiagnosed prior to ILD diagnosis, with chronic obstructive pulmonary disease suspected in 39.2% of them. The most frequent comorbidities were anxiety (16.9%) and gastroesophageal reflux (15.5%). Although 77.8% of patients were receiving treatment for ILD at survey date, 15.6% of patients had never been prescribed treatment for ILD.

**Conclusions:**

This real-world study expands our understanding of patients, diagnostic delays and treatment gaps experienced by patients diagnosed with PPF in Europe. There was a mean delay of 15.5 months between first ILD symptoms and ILD diagnosis. Given the progressive nature of PPF, diagnostic delay may lead to poor outcomes, including shorter survival.

**Trial registration:**

N/a.

**Supplementary Information:**

The online version contains supplementary material available at 10.1186/s12931-024-02995-9.

## Background

Interstitial lung diseases (ILDs) are a large and heterogeneous group of parenchymal pulmonary disorders, some of which have a progressive fibrosing phenotype [1, 2]. Idiopathic pulmonary fibrosis (IPF), the archetypal progressive fibrosing ILD and the most common idiopathic interstitial pneumonia, is characterised by chronic progressive fibrosis, worsening of lung function and dyspnoea [1, 3], and has a life expectancy of approximately 3–5 years after diagnosis if left untreated [4–6]. Approximately one third of patients with fibrosing ILDs other than IPF may experience disease progression, defined as progressive pulmonary fibrosis (PPF) [1, 7, 8].

There are currently two licensed therapies for IPF, pirfenidone and nintedanib, which have been shown to slow the rate of lung function decline [9–13]. Nintedanib has also been approved for the treatment of progressive fibrosing ILDs other than IPF (i.e., PPF), and ILD associated with systemic sclerosis (SSc-ILD) [10, 11]. However, the rate of patient access to treatment is mixed. In the INSIGHTS-ILD registry of patients in Germany with PPF (excluding IPF), 45.8% were receiving nintedanib and 2.1% were receiving pirfenidone [14], whereas in the US-based ILD-PRO registry of patients with PPF (also excluding IPF), 19.8% were receiving nintedanib and 3.6% were receiving pirfenidone [15].

PPF is characterised by worsening respiratory symptoms, decline in lung function, radiographic progression and early mortality despite appropriate management, and may have a clinical course similar to IPF [3, 16, 17]. Regardless of the underlying condition, PPF occurs through similar mechanisms of self-sustained dysregulated cell repair, fibroblast proliferation and alveolar dysfunction [3, 17]. However, patients with certain types of fibrosing ILDs are more prone to progression than others: it has been estimated that PPF can develop in 53% of patients with unclassifiable ILD (uILD), 40% of patients with SSc-ILD, 32% of patients with rheumatoid arthritis-associated ILD (RA-ILD), and in 21% of patients with fibrotic hypersensitivity pneumonitis [7].

In addition, the ILD disease course may be heterogeneous, with different rates and patterns of progression [18]. PPF has been shown to be associated with significantly higher healthcare resource utilisation (HCRU) compared with non-progressive fibrosing ILDs [19, 20]. Several studies have shown that patients with PPF required more follow-up visits, hospitalisations, days in hospital and laboratory/imaging tests than patients with ILD that is non-progressive or slowly progressing [19]. Indirect costs such as loss of work productivity, job losses or early retirement were also higher for patients with PPF than those with the non-progressive phenotype [19, 20].

There is a good understanding of the epidemiology and burden of IPF as well as its frequent association with other diseases and comorbidities. The presence of these comorbidities may cause a delay in diagnosis and interfere with the disease course, thus impacting patient prognosis [21, 22]. However, little is known about the burden of PPF, the patient journey to diagnosis, the prevalence of associated comorbidities and its impact on patients’ quality of life [23]. Likewise, there is a lack of understanding of the best approach to diagnose, manage and treat PPF. Therefore, this study aimed to investigate the burden, diagnosis, referral patterns and current management practices of PPF in a European real-world setting.

Note on terminology: this study was devised, and the data collected, before publication of the updated 2022 guidelines for IPF and PPF by the American Thoracic Society (ATS), the European Respiratory Society (ERS), the Japanese Respiratory Society (JRS), and the Latin American Thoracic Association (ALAT), which provided a definition of PPF [1]. No specific definition of progression was used, but the disease was required to have a progressive phenotype in the opinion of the treating physician. However, for clarity, the term PPF is used throughout this manuscript.

## Methods

### Study design

Data were drawn from the Adelphi Real World PPF-ILD Disease Specific Programme (DSP)^TM^, a cross-sectional survey of physicians (pulmonologists, rheumatologists or internal medicine specialists [France only]) in Europe (France, Germany, Italy, Spain, the United Kingdom [UK]) who had access to medical history data [24, 25]. Full details of the DSP methodology have been published previously [24, 26].

**Fig. 1:**
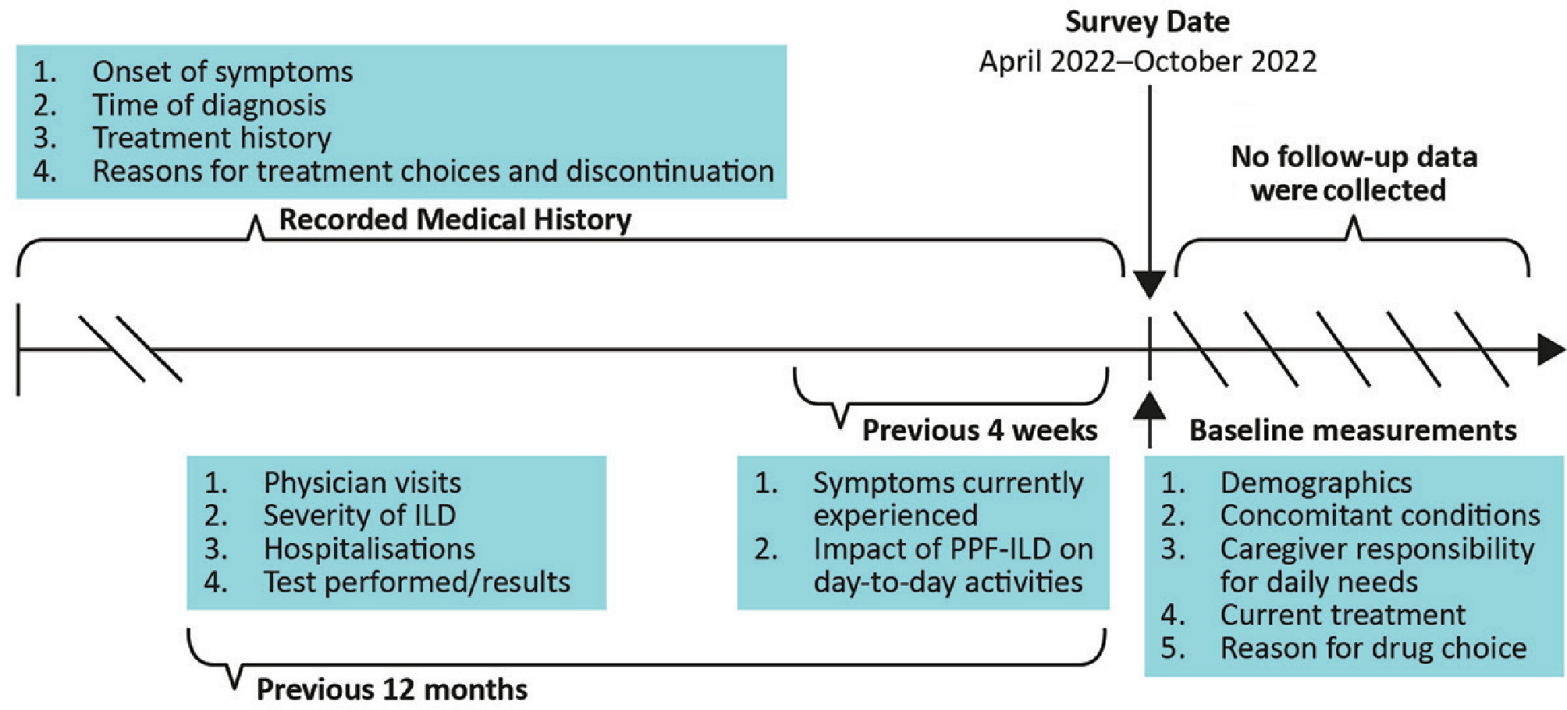
Schematic of information collection in the PPF-ILD DSP study. DSP, Disease Specific Programme; ILD, interstitial lung disease; PPF, progressive pulmonary fibrosis

Physicians captured data on patient demographics, clinical characteristics, symptom burden and impact, patient management, treatment utilisation and decision-making in routine care. All information was recorded at a single point in time during the period April to October 2022 using available medical history or a specified recall period for physicians (Fig. 1); no follow-up information was collected.

### Data collection

Participating physicians completed surveys, which provided general information on management, referrals, usage, and awareness of antifibrotics and attitudes towards PPF. Physicians were instructed to complete patient record forms (PRFs) for up to 12 consecutively consulted patients with a physician-confirmed ILD diagnosis with a progressive phenotype. Each record included details regarding patient demographics, clinical characteristics (including physician-perceived disease severity, or according to Goh’s criteria [27] for SSc-ILD only, at different stages in the patient’s journey), symptom burden and treatment history. Prospective PRFs were completed by the physician for qualifying patients as and when they consulted the physician (main DSP sample). Retrospective PRFs were collected to augment the size of the main DSP sample and were completed by additional physicians (i.e., those who had not completed surveys as part of the main DSP sample) for their last 4–6 qualifying patients. Physician-reported disease severity (mild, moderate, severe) and progression (improving, stable, progressing) were based on physician opinion and not predefined. The terms ‘[progressive fibrosing] PF-ILD’ and ‘[connective tissue disease] CTD-ILD’ were used in the physician surveys and PRFs.

### Study population

Patients were eligible for inclusion if they were aged over 18 years, had a physician-confirmed diagnosis of ILD and presented with a progressive phenotype (PPF), as determined by the reporting physician. No clinical definition of a progressive phenotype was pre-specified as the study was performed prior to publication of the current ATS/ERS/JRS/ALAT guidelines [1]; diagnoses therefore included the following: idiopathic non-specific interstitial pneumonia (iNSIP), fibrotic hypersensitivity pneumonitis, uILD, SSc-ILD, RA-ILD, polymyositis/dermatomyositis-ILD and Sjögren’s-associated ILD. Patients with IPF were excluded.

Target physicians were identified in the respective European countries from public lists of healthcare professionals (HCPs). Physicians were pulmonologists, rheumatologists, or internal medicine specialists. To be included in the study, pulmonologists were required to see at least four different types of qualifying ILDs in a typical month, internal medicine specialists were required to see at least four qualifying patients with ILD in a typical month, and rheumatologists were required to see at least two different types of CTD-ILD (RA-ILD, SSc-ILD, polymyositis/dermatomyositis-ILD or Sjögren’s-associated ILD) in a typical month.

### Data analyses

As the primary research objective was descriptive in nature (i.e., no a priori hypotheses specified), the available sample size of physicians and patients was driven by the DSP data collection methodology. Formal sample size calculations were not applicable, and therefore not performed, meaning that the sample size impacted the precision of any estimates (see Supplementary Methods in Additional File 1 for details; Table S1–S2). Descriptive analyses were undertaken by Adelphi Real World and conducted in UNICOM^®^ Data Collection Survey Reporter (UNICOM Global, Inc, Mission Hills, CA, USA). Descriptive statistics were used to characterise demographics, clinical characteristics, symptom burden/impact, treatment history, patient ILD milestones and consultation history, hospitalisation and HCRU. For age-based patient ILD milestones (e.g., age at first HCP visit, age at first symptom, age at diagnosis), only patients with a value recorded for each of the milestones were included. Bivariate analyses (Fisher’s exact test, T-test) were used to compare demographic data for patients with CTD-ILD and other types of ILD.

### Ethics statement

Data were collected by local fieldwork partners, and both physician and patient data were de-identified prior to receipt by Adelphi. The study received Pearl Institutional Review Board ethical exemption (22-ADRW-135) and was conducted adhering to European Pharmaceutical Market Research Association guidelines and Adelphi Real World standard operating procedures.

## Results

### PPF-ILD DSP survey sample

Two hundred and sixty-five physicians participated in the survey and completed PRFs for 1335 patients with a physician-confirmed ILD diagnosis with PPF (Table 1). The sample was composed of 66.1% pulmonologists, 30.9% rheumatologists and 3.1% internal medicine specialists (only recruited in France). The largest group of patients were based in Germany (24.6%) and the smallest in the UK (13.8%) (Table S3). The ratio of patients to physicians was greatest in France (6.9) and lowest in the UK (3.7).

**Table 1:** Study sample size

Physician specialty	Physician surveys*	Patient record forms
Total	265	1,335
Pulmonologist	175	790
Rheumatologist	82	484
Internal medicine	8	61

*Completed by all physicians prospectively completing patient record forms for the survey

### Patient demographics

The mean (standard deviation [SD]) age of patients at the survey date was 60.4 (11.6) years, 91.2% were white, 58.1% were female, 44.0% were non-smokers, and mean (SD) body mass index was 25.3 (3.9) kg/m^2^ (Table 2). Few patients (5.4%) had a family history of ILD. At the survey date, 25.2% of patients were in full-time employment. Of those not in full-time employment (*n* = 806), 18.9% were unable to work full-time due to their ILD (either working part-time, retired, on long-term sick leave or unemployed).

**Table 2:** Patient demographics by type of PPF

		Type of PPF						
		Other ILDs			CTD-ILD			
	Total (*N* = 1,335)	iNSIP (*n* = 263)*	Fibrotic HP (*n* = 225)	uILD (*n* = 133)	SSc-ILD (*n* = 263)	RA-ILD (*n* = 269)	PM/DM-ILD (*n* = 83)	SS-ILD (*n* = 99)
Age, mean (SD)	60.38	62.50	62.08	66.41	56.14	61.06	53.83	57.76
	11.59	11.09	11.68	12.02	10.56	10.56	11.68	10.73
BMI, mean (SD)	25.31	25.53	26.29	25.88	24.07	25.42	25.41	24.58
	3.89	3.24	4.00	4.20	3.99	4.08	3.57	3.39
Female sex, *n* (%)	776	126	68	56	223	166	53	84
	58.13	47.90	30.22	42.10	84.79	61.71	63.86	84.84
Ethnicity								
White/Caucasian	1,217 (91.16)	245 (93.16)	216 (96.00)	121 (90.98)	237 (90.11)	251 (93.31)	61 (73.49)	86 (86.87)
Asian (Indian subcontinent)	11 (0.82)	0 (0.00)	1 (0.44)	1 (0.75)	4 (1.52)	0 (0.00)	2 (2.41)	3 (3.03)
Asian (other)	5 (0.37)	0 (0.00)	0 (0.00)	1 (0.75)	2 (0.76)	0 (0.00)	2 (2.41)	0 (0.00)
Hispanic/Latino	30 (2.25)	5 (1.90)	3 (1.33)	3 (2.26)	6 (2.28)	6 (2.23)	5 (6.02)	2 (2.02)
Middle Eastern	25 (1.87)	1 (0.38)	1 (0.44)	4 (3.01)	7 (2.66)	5 (1.86)	5 (6.02)	2 (2.02)
Mixed race	10 (0.75)	8 (3.04)	0 (0.00)	0 (0.00)	0 (0.00)	1 (0.37)	0 (0.00)	1 (1.01)
Afro-Caribbean	28 (2.10)	3 (1.14)	3 (1.33)	2 (1.50)	5 (1.90)	3 (1.12)	7 (8.43)	5 (5.05)
South-East Asian	9 (0.67)	1 (0.38)	1 (0.44)	1 (0.75)	2 (0.76)	3 (1.12)	1 (1.20)	0 (0.00)
Other	0 (0.00)	0 (0.00)	0 (0.00)	0 (0.00)	0 (0.00)	0 (0.00)	0 (0.00)	0 (0.00)
Smoking status								
Current smoker	98 (7.34)	25 (9.51)	19 (8.44)	12 (9.02)	11 (4.18)	20 (7.43)	5 (6.02)	6 (6.06)
Ex-smoker	612 (45.84)	128 (48.67)	104 (46.22)	59 (44.36)	107 (40.68)	140 (52.04)	32 (38.55)	42 (42.42)
Never smoked	588 (44.04)	103 (39.16)	99 (44.00)	60 (45.11)	132 (50.19)	99 (36.80)	46 (55.42)	49 (49.49)
Don’t know	37 (2.77)	7 (2.66)	3 (1.33)	2 (1.50)	13 (4.94)	10 (3.72)	0 (0.00)	2 (2.02)
Employment status								
Working full-time	336 (25.17)	70 (26.62)	64 (28.44)	20 (15.04)	60 (22.81)	65 (24.16)	28 (33.73)	29 (29.29)
Working part-time	145 (10.86)	20 (7.60)	19 (8.44)	6 (4.51)	42 (15.97)	27 (10.04)	14 (16.87)	17 (17.17)
On long-term sick leave	76 (5.69)	10 (3.80)	14 (6.22)	7 (5.26)	24 (9.13)	13 (4.83)	7 (8.43)	1 (1.01)
Homemaker	161 (12.06)	37 (14.07)	15 (6.67)	11 (8.27)	49 (18.63)	29 (10.78)	5 (6.02)	15 (15.15)
Student	1 (0.07)	0 (0.00)	0 (0.00)	0 (0.00)	0 (0.00)	0 (0.00)	1 (1.20)	0 (0.00)
Retired	517 (38.73)	114 (43.35)	98 (43.56)	79 (59.40)	64 (24.33)	116 (43.12)	17 (20.48)	29 (29.29)
Unemployed	68 (5.09)	7 (2.66)	6 (2.67)	10 (7.52)	15 (5.70)	13 (4.83)	10 (12.05)	7 (7.07)
Don’t know	31 (2.32)	5 (1.90)	9 (4.00)	0 (0.00)	9 (3.42)	6 (2.23)	1 (1.20)	1 (1.01)

*Total for iNSIP for age is *n* = 262

BMI, body mass index; CTD, connective tissue disease; DM, dermatomyositis; HP, hypersensitivity pneumonitis; iNSIP, idiopathic non-specific interstitial pneumonia; ILD, interstitial lung disease; PM, polymyositis; PPF, progressive pulmonary fibrosis; RA, rheumatoid arthritis; SD, standard deviation; SS, Sjögren’s; SSc, systemic sclerosis; uILD, unclassifiable interstitial lung disease

Numerically, patient demographics as defined by country or physician specialty were similar (Tables S4–S5). The proportion of patients either working part-time, retired, on long-term sick leave or unemployed due to their ILD was comparable with the main survey population (20.1%) for both patients with CTD-ILD and other types of ILD (21.9% and 18.3%, respectively; Fisher’s exact test *p* < 0.2387). However, patients with CTD-ILD were significantly younger than those with other types of ILD (58.0 [SD 11.0] and 62.3 [SD 11.7] years, respectively; t-test *p* < 0.0001), and a greater proportion were female (73.7% and 40.3%, respectively; t-test *p* < 0.0001) (Table 2, Table S6).

### Clinical characteristics

#### Disease severity

**Fig. 2:**
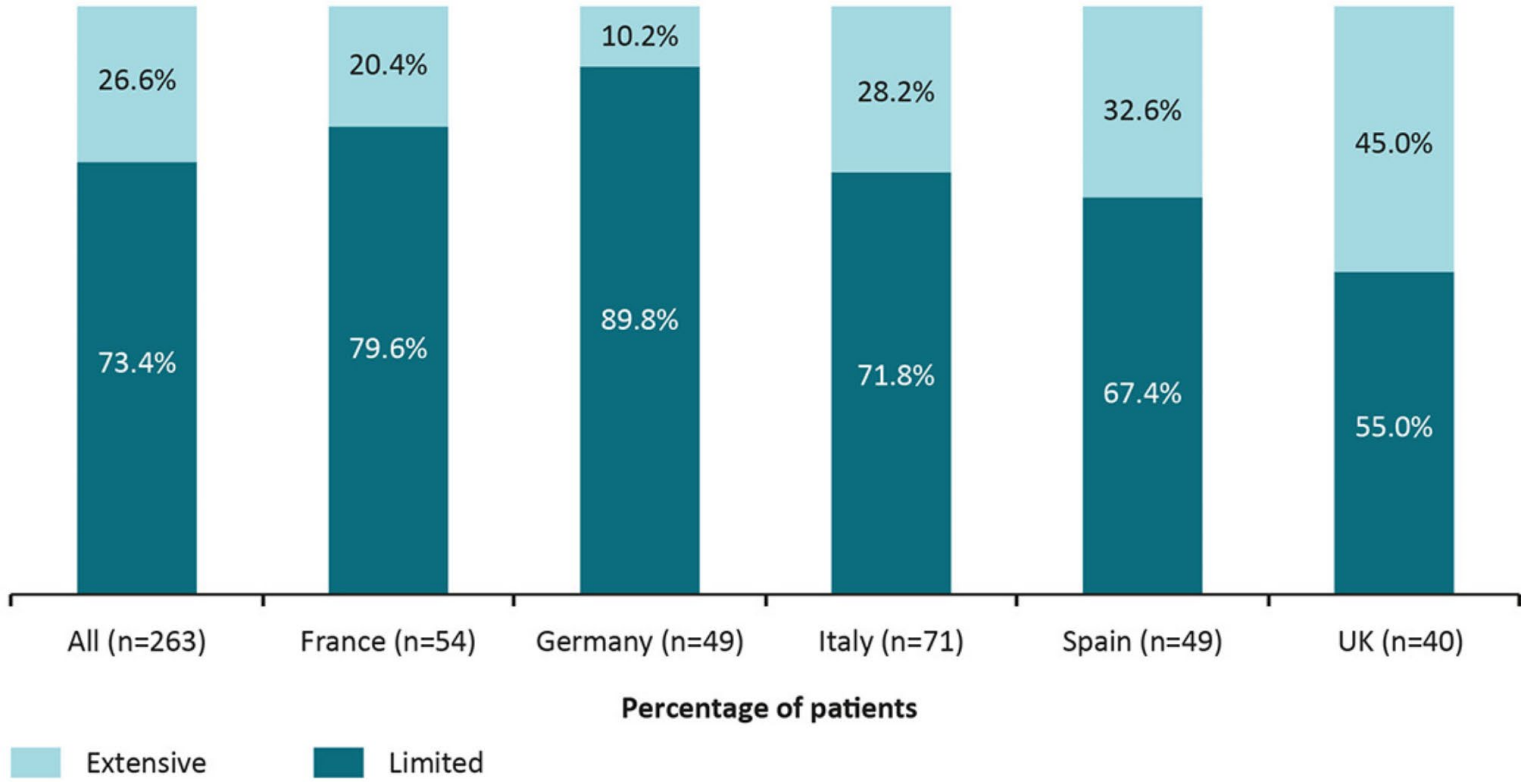
Physician-rated severity of SSc-ILD according to Goh’s criteria. ILD, interstitial lung disease; SSc, systemic sclerosis; UK, United Kingdom

Based on physician-reported disease severity, more patients were reported to have moderate ILD (47.7%) at survey date than mild (42.3%) or severe ILD (10.0%). The proportion of patients with severe ILD varied by reporting physician (rheumatologists 5.8%, pulmonologists 12.5%), country (Germany 0.6%, Spain 15.6%) and type of ILD (Sjögren’s-associated ILD 3.0%, polymyositis/dermatomyositis-associated ILD 14.5%) (Figure S1). For patients with SSc-ILD (*n* = 263), most patients were classed as having limited disease (73.4%) compared with extensive disease (26.6%) for physician-reported severity of ILD according to Goh’s criteria [27] at survey date (Fig. 2).

#### Disease progression

**Fig. 3:**
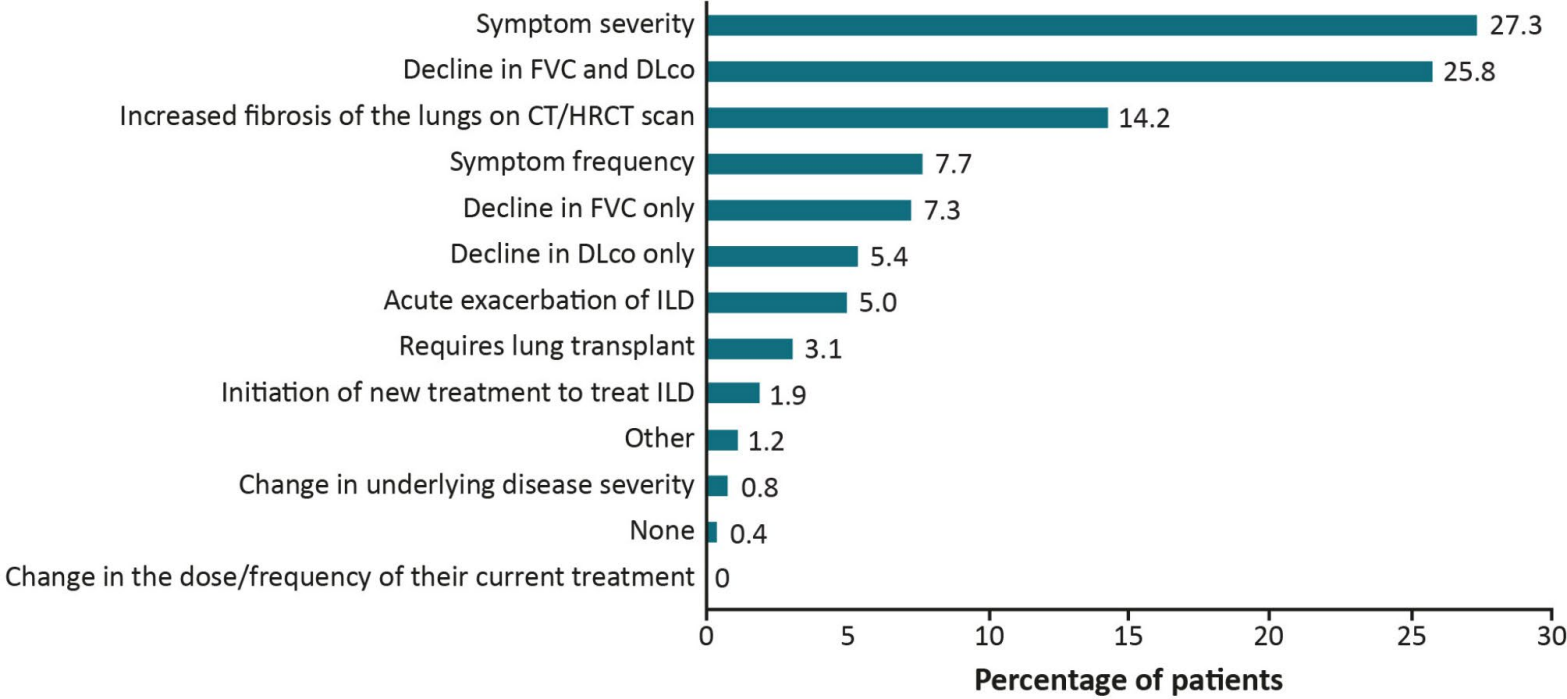
Physician-reported reasons for disease progression in the last 12 months. CT, computed tomography; DLco, diffusing capacity of the lungs for carbon monoxide; FVC, forced vital capacity; HRCT, high-resolution CT; ILD, interstitial lung disease

Overall, 19.5% of patients were reported as progressing in the 12 months prior to survey date (moderately progressing 16.6%, severely progressing 2.9%), 52.2% were stable, 26.1% were improving and 2.3% were too early to tell. For those patients deemed to have progression, physicians provided one main reason for their assessment; the most frequent were worsening symptom severity (27.3%), decline in lung function (forced vital capacity [FVC] and diffusing capacity of the lungs for carbon monoxide [DLco]; 25.8%) or increased fibrosis on imaging (14.2%) (Fig. 3). Reasons for progression varied by country and physician specialty (Tables S7 & S8). For example, progression due to symptom severity ranged between 15.0% in Spain to 42.2% in the UK. Progression was reported based on symptom severity for 26.4% of patients seeing a pulmonologist and 32.6% of patients seeing a rheumatologist, and based on decline in FVC or DLco for 27.4% of patients seeing a pulmonologist and 17.4% of patients seeing a rheumatologist. Reasons for progression varied by type of ILD, with symptom severity (30.4%) or increased extent of fibrosis (19.6%) mostly reported for uILD; for RA-ILD, progression was mostly reported due to decline in lung function (29.2%) or symptom severity (25.0%) (Table S9).

Of those patients diagnosed with ILD less than 1 year before survey date, 16.8% of patients were reported to have moderately or severely progressing disease compared with 21.0% of patients diagnosed more than 1 year before (Figure S2). Physicians expected that over the next 12 months from the survey date, progression would stabilise for more than half (61.4%) of all patients. This ranged from 48.1% of patients with uILD to 70.3% of patients with RA-ILD. Expected progression in the next 12 months from the survey date based on physician perspective was aligned with clinical parameters, with patients considered as progressing presenting with lower FVC and DLco (Figure S3).

#### Comorbidities

A total of 61.8% of patients had at least one comorbidity; the mean (SD) number of comorbidities per patient was 1.3 (1.5) and ranged from 0 to 12. The most frequent physician-reported comorbidities were anxiety (16.9%), gastroesophageal reflux (15.5%), depression (13.4%), diabetes without chronic complications (10.0%) and pulmonary hypertension (9.0%). There was some variation in comorbidities reported according to country, physician specialty and type of ILD (Tables S10–S12). For example, anxiety was most frequently reported in Italy (24.7%) and France (22.5%) (lowest in Germany at 3.1% of patients) and pulmonary hypertension was reported for 14.5% of patients with SSc-ILD and 6.0% each of patients with RA-ILD and polymyositis/dermatomyositis-ILD.

### Treatment

Mean (SD) age at initial treatment was 57.8 (12.3) years (*n* = 1,000). A total of 77.8% of patients were receiving treatment for their ILD at the survey date; of those, low-dose prednisone (41.5%), nintedanib (30.9%) and mycophenolate (19.1%) were the most frequent treatments (Table 3). Of those prescribed low-dose prednisone (*n* = 431), 21.3% were prescribed low-dose prednisone only and 78.7% were prescribed low-dose prednisone plus other treatment; of those prescribed nintedanib (*n* = 321), 34.0% were prescribed nintedanib only and 66.0% were prescribed nintedanib plus other treatments; of those prescribed mycophenolate (*n* = 198), 16.2% were prescribed mycophenolate only and 83.8% were prescribed mycophenolate plus other treatments.

**Table 3:** Patient treatment history

Patient treatment	
Patients currently prescribed a treatment for their ILD, *n*	1335
Yes	1,039 (77.83)
No, but previously prescribed treatment	88 (6.59)
No, has never been prescribed treatment	208 (15.58)
Duration of current ILD treatment, *n*	705
Mean, years (SD)	1.64 (3.88)
Treatment prescribed to treat underlying autoimmune disease, *n*	586
Currently prescribed treatment for ILD, *n*	1039
Low-dose prednisone	431 (41.48)
Nintedanib	321 (30.90)
Mycophenolate	198 (19.06)
Methotrexate	152 (14.63)
Cough suppressants	133 (12.80)
Rituximab	130 (12.51)
Inhaled corticosteroids	128 (12.32)
High-dose prednisone	126 (12.13)
Oxygen therapy	94 (9.05)
Azathioprine	92 (8.85)
Pirfenidone	81 (7.80)
Other	42 (4.04)
Tocilizumab	34 (3.27)
Cyclophosphamide	24 (2.31)
Baricitinib	12 (1.15)
Tacrolimus	10 (0.96)
Upadacitinib	9 (0.87)
Cyclosporine	4 (0.38)
Tofacitinib	4 (0.38)
Peficitinib	0 (0.00)
Apremilast	0 (0.00)
Next course of action for inadequate response to current treatment	1039
Switch product and replace	195 (18.77)
Increase dose	178 (17.13)
Add a product to current regimen	172 (16.55)
Pulmonary rehabilitation	122 (11.74)
Oxygen support	80 (7.70)
Lung transplant	52 (5.00)
Reduce dose	36 (3.46)
Stem cell transplant	0 (0.00)
Other	19 (1.83)
Don’t know	190 (18.29)
Reason for never prescribing treatment for ILD	208
Profile is manageable without treatment	101 (48.56)
Symptoms not severe enough to warrant treatment	43 (20.67)
Diagnosed too recently	36 (17.31)
Patient request	34 (16.35)
Patient concerns over side effects	33 (15.87)
Due to the underlying autoimmune disease	10 (4.81)
Lack of information in current treatment guidelines	10 (4.81)
Lack of evidence of the efficacy of current drug treatments available	9 (4.33)
No approved therapies specifically for ILD	8 (3.85)
Waiting for new drugs/products to be approved by regulatory bodies	7 (3.37)
Disease-modifying treatment considered last option of care	3 (1.44)
Medication is not covered by the patient’s insurance	2 (0.96)
Other	14 (6.73)

Data are n (%) unless otherwise indicated

ILD, interstitial lung disease; SD, standard deviation

For those with available data (*n* = 705; 67.9%), mean (SD) current treatment duration was 1.6 (3.9) years at survey date. The proportion of patients currently prescribed treatment varied by country (ranging from Germany 88.4% to France 71.7%), physician specialty (pulmonologists 60.0%, rheumatologists 43.2%) and type of ILD (polymyositis/dermatomyositis-associated ILD 88.0%, uILD 54.1%) (Tables S13–S15).

If current treatment was deemed to be inadequate as judged by the treating physician, the most frequently reported next steps included switching to a different treatment (18.8%), increasing the dose (17.1%), or adding a treatment to the existing regimen (16.6%), although 18.3% of physicians did not know what the next course of action would be (Table 3). A numerically higher proportion of physicians in the UK would switch treatment compared with physicians in Germany (29.6% and 13.8%, respectively) (Figure S4).

Some patients (6.6%) were not receiving treatment for their ILD at survey date but had previously been treated (Table 3). Of these, 44.3% had discontinued low-dose prednisone, 33.0% discontinued high-dose prednisone, and 20.5% discontinued nintedanib. The main reasons given by physicians for discontinuing treatment were side effects (26.1%) and poor adherence to prescribed treatment (23.9%). Overall, 15.6% of patients had never been prescribed treatment for their ILD (Table 3). The most common reason for never receiving treatment as reported by physicians was the disease being considered manageable without treatment (48.6%), which was consistent across countries, physician specialty and type of ILD.

### Patient journey

#### Clinical milestones

**Fig. 4:**
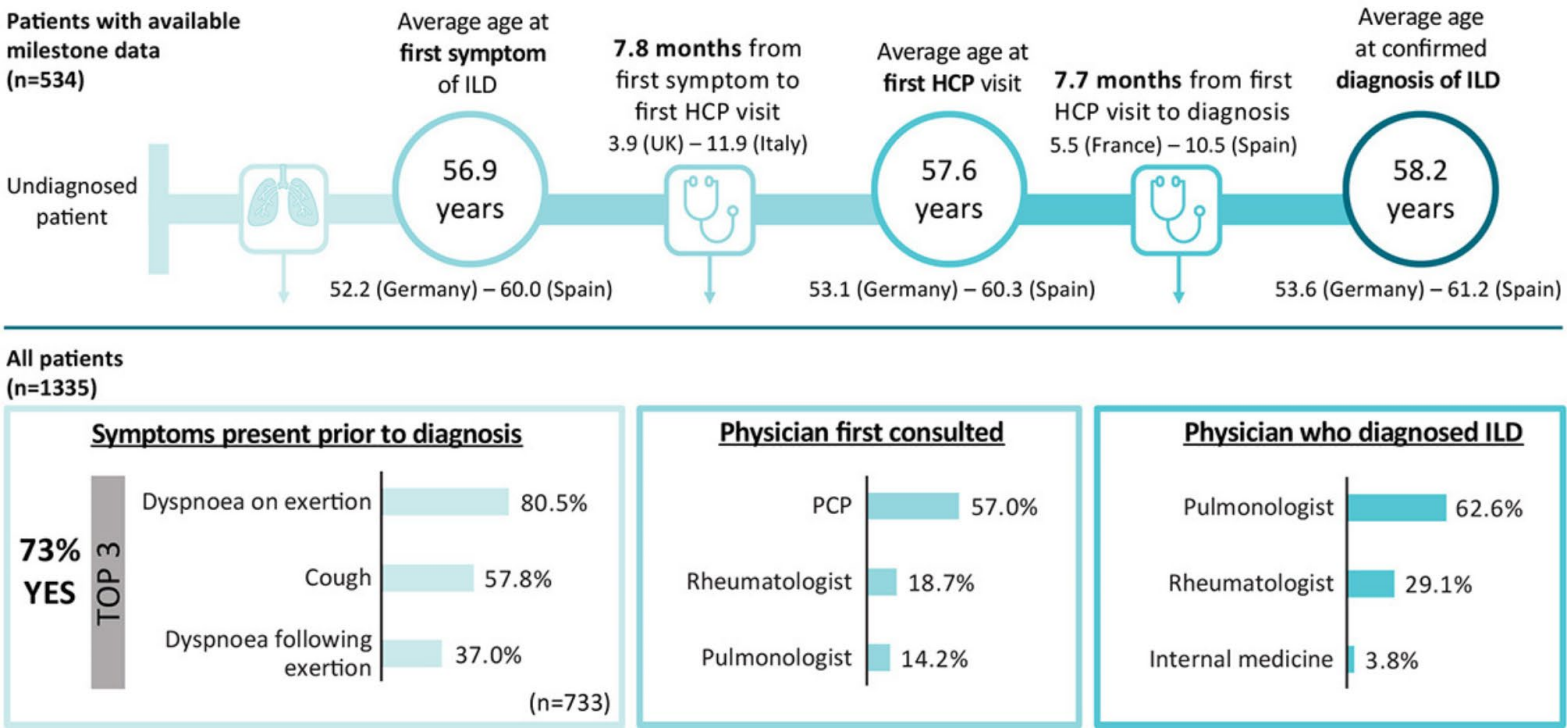
Patient journey for ILD in Europe. HCP, healthcare professional; ILD, interstitial lung disease; PCP, primary care physician

A total of 534 patients (40.0%) had data available regarding their diagnostic journey (Fig. 4). For these patients, the mean (SD) age at first symptom of ILD was 56.9 (12.0) years, mean age at first HCP visit was 57.6 (12.1) years, and mean age at confirmed ILD diagnosis was 58.2 (12.2) years (Tables S16–S18). In total, there was an average of 15.5 months between first ILD symptoms and ILD diagnosis.

Patient journey varied by country, reporting physician and type of ILD. For example, patients in Germany were generally younger across all milestones (e.g., mean age [SD] 52.2 [9.5] years at first symptom of ILD compared with 60.0 [11.2] years for patients in Spain). Pulmonologists reported that patients were older at first ILD symptom (mean [SD] 59.4 [11.5] years) and first HCP visit (60.2 [11.5] years) than those reported by rheumatologists (54.6 [11.2] years and 54.9 [11.2] years, respectively). Patients with Sjögren’s-associated ILD had a confirmed diagnosis of ILD at a mean (SD) age of 48.2 (14.0) years, whereas patients with SSc-ILD were mean age 62.8 (12.4) years at diagnosis.

There was a mean (SD) delay of 7.8 (22.7) months between first ILD symptom and first HCP visit, with a further delay of 7.7 (12.8) months between first HCP visit and ILD diagnosis. This varied by country, with mean (SD) delay between first HCP visit and ILD diagnosis of 5.5 (7.2) months in France and 10.5 (14.5) months in Spain. Patients seeing a pulmonologist generally had a longer mean delay between first HCP visit and confirmed diagnosis of ILD (mean [SD] 9.1 [14.0] months). The mean delay between first HCP visit and ILD diagnosis varied between type of ILD; however, there was also wide variation within each type of ILD.

#### Symptom burden and diagnosis

Of those patients reporting or presenting with symptoms at the survey date (*n* = 1,007), 72.8% of them experienced symptoms of ILD prior to a confirmed diagnosis. The most frequent symptoms experienced prior to an ILD diagnosis were dyspnoea on exertion (80.5%), cough (57.8%) and dyspnoea following exertion (37.0%). Among the 1,007 patients reporting symptoms in the 4 weeks prior to survey date, the most frequent were dyspnoea on exertion (78.4%) and cough (49.4%).

Overall, 232 patients (17.4%) were diagnosed with another condition prior to ILD diagnosis; of these, chronic obstructive pulmonary disease (COPD) was suspected or investigated in 39.2% of patients and asthma in 22.4% of patients. Rates for conditions suspected or investigated prior to ILD diagnosis varied by country, reporting physician specialty and type of ILD (Tables S19–S21). In Germany (*n* = 54), COPD was the most frequently suspected or investigated condition (77.8%), whereas in Spain (*n* = 52), asthma (32.7%) was the most frequent. For pulmonologists (*n* = 164), COPD (36.6%), asthma (25.6%) and heart failure (10.4%) were the most frequently suspected or investigated conditions. For rheumatologists (*n* = 60), these were COPD (48.3%), anxiety (21.7%) and bronchitis (20.0%). COPD was the most frequently suspected or investigated condition across the different types of ILD, except for fibrotic hypersensitivity pneumonitis (*n* = 55; asthma, 43.6%) and SSc-ILD (*n* = 31; anxiety and COPD, both 25.8%).

#### Consultation and referral history

**Fig. 5:**
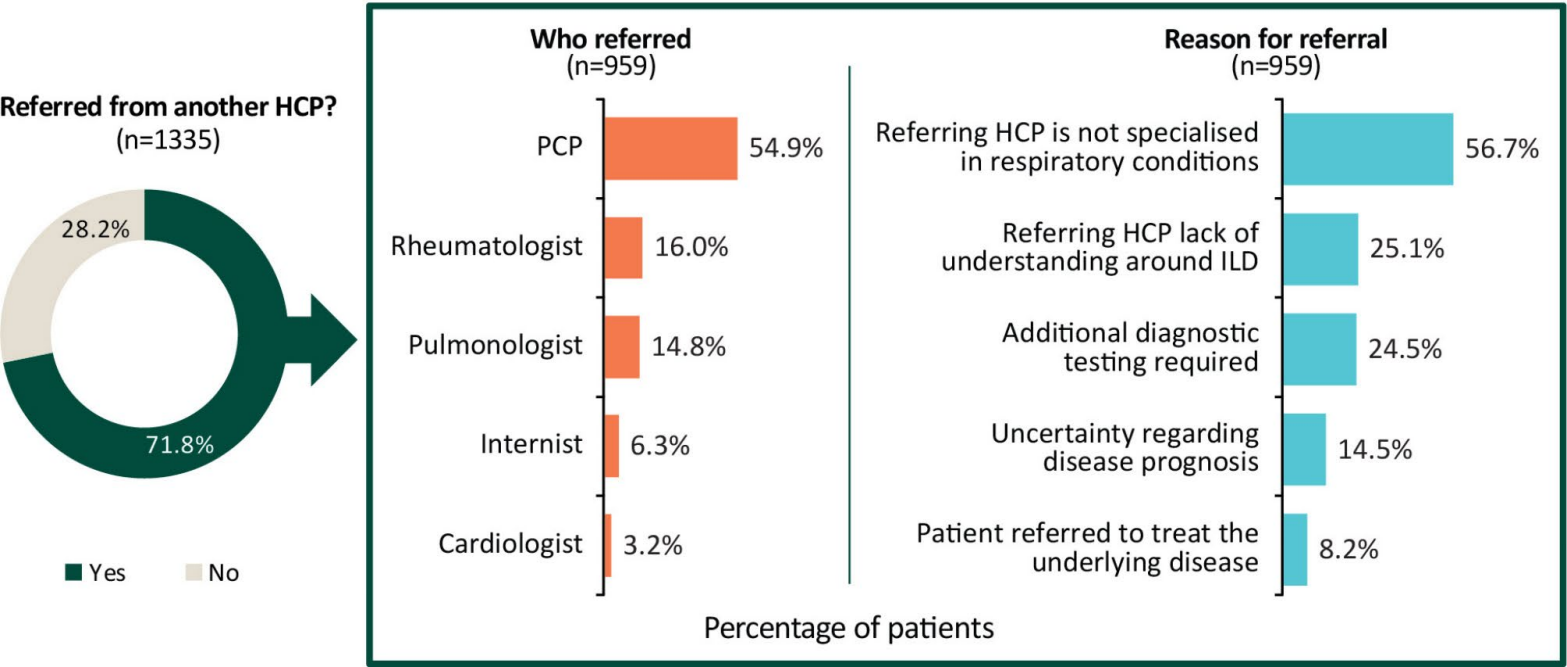
Physician-reported patient referral. HCP, healthcare professional; ILD, interstitial lung disease; PCP, primary care physician

Most patients first consulted a primary care physician (57.0%) regarding their ILD symptoms. A total of 71.8% of patients were referred from another HCP, most frequently by a primary care physician (54.9%) (Fig. 5). The most common reasons for referral were that the referring HCP did not specialise in respiratory conditions (56.7%), a lack of understanding around ILD (25.1%) and the need for additional diagnostic testing (24.5%). A pulmonologist was the diagnosing physician in 62.6% of cases, and additional physicians were involved in the diagnosis of 24.9% of all cases. According to the physician surveys (*n* = 265), 83.4% of respondents were part of a multidisciplinary team, and within those teams most had a role in diagnosis and ongoing management (91.4%). This varied across countries, with the use of a multidisciplinary team for diagnosis reported in 40.6% of patients in Italy and 4.0% of patients in Germany.

## Discussion

This PPF-ILD survey adds to our understanding about care and management of patients diagnosed with ILD with PPF in Europe, in particular, the different experiences of patients with various types of ILD. Consistent with previous survey studies of patients with ILD, results showed that there was a delay of 15.5 months in diagnosis of ILD after first symptoms, including dyspnoea on exertion and cough [28, 29].

While there is a good understanding of the epidemiology and burden of IPF, little is known about the burden of PPF, the prevalence of comorbidities, and the patient journey to diagnosis. Diagnosis of ILD in general is often delayed, as is recognition of PPF, with PPF relying on evidence of progression via regular monitoring [1].

Over 17% of the patients in this study were misdiagnosed before ILD diagnosis. The rate of misdiagnosis in our study was lower than expected compared with existing literature; for instance, in a study of registered members of the Pulmonary Fibrosis Foundation, 56% of patients with ILD had experienced at least one misdiagnosis [28].

On average, most patients had at least one comorbidity reported in the survey, with anxiety (16.9%), gastroesophageal reflux (15.5%) and depression (13.4%) the most frequent overall. While there is limited published data available for patients with PPF, in the EMPIRE registry of patients with IPF in Central and Eastern European countries, 91% of patients reported at least one comorbidity, and more than 38% reported four or more comorbidities [22]. The presence of multiple comorbidities at enrolment into the registry was associated with worse survival [22], similar to findings in patients with IPF at a tertiary referral centre [30]. The current study adds to the limited existing literature on comorbidities, highlighting the importance of identifying and treating comorbidities in PPF.

Given the progressive nature of PPF, understanding the patient pathway for different types of ILD and in difference countries is essential, as any delay in diagnosis and treatment may lead to a poorer prognosis. In patients with IPF, a diagnostic delay of more than a year is associated with worsened prognosis and quality of life [31]. We found the main reasons for patient referral included the lack of specialty in respiratory conditions or lack of understanding of ILD, aligning with a retrospective analysis of an ILD referral tertiary academic centre in which 51% of patients with ILD were referred from general practice [32]. With a total mean (SD) of 15.5 (26.6) months between a patient first experiencing symptoms and an ILD diagnosis, and as most patients (57%) presented in primary care, there is a need for physician education on disease behaviour in PPF and for regular monitoring for progression to reduce the delay between first presentation with ILD symptoms and prompt referral to expert centres for diagnosis. Education and disease awareness programmes are also needed for patients and the general public to help encourage people to recognise symptoms and seek medical advice.

Nearly 20% of patients were reported as progressing, based on worsening symptom severity, decline in lung function or increased fibrosis. In the current study, physicians reported decline in FVC percent predicted, worsening of symptoms and decline in DLco percent predicted as the key attributes for defining progression, in alignment with the ATS/ERS/JRS/ALAT guidelines [1]. Progression according to increased pulmonary fibrosis on high-resolution computed tomography (HRCT) was the least reported category out of the three; however, only one criterion for progression could be selected, which may have affected the results. Differences were noted by physician specialty, with more pulmonologists reporting a decline in FVC as the most important factor defining progression than rheumatologists, and rheumatologists using DLco more often than FVC. This highlights the importance of multidisciplinary management, particularly for patients with CTD-ILD [32, 33], given evaluation of ILD with pulmonary function tests is an expertise mainly of pulmonologists. In addition, FVC decline has been shown to have value as a predictor of mortality in both patients with IPF and with PPF [34].

Although this was a study on PPF, 52% of patients had progression that was reported as stable over the previous 12 months from the survey date, and 42% of patients were deemed to have ‘mild’ disease severity at survey date based on physician opinion. This may be due to the physician’s expectation that they can at best stabilise progression for a short period of time for the most optimistic outcome possible. In a similar survey in IPF, patients with physician-reported mild or moderate disease were more likely to be classified as stable or improving than those with severe disease according to FVC data [35]. The authors of the IPF study [35] concluded that physician-reported severity was misaligned with severity as determined by FVC, potentially due to unfamiliarity with the disease or inadequately assessing symptoms.

For those patients predicted to have ‘stable’ disease in the next 12 months from the survey date, this may be due to physicians expecting to observe a treatment effect. As no definitions of severities were included in this study, analysis is restricted by the subjective nature of physician-determined ‘stable’ or ‘mild’ disease. The use of ‘mild’, ‘moderate’ or ‘severe’ as undefined terms to describe disease state have been used in IPF [36, 37]. In a chart survey of patients with IPF, between 26% and 44% of patients were perceived to have mild disease and 10–16% were perceived to have severe disease by their physician [37]. Such findings are comparable with results from this study, and highlight potential disparities between physicians’ interpretation of severity versus known burden and mortality of PPF.

This study also found around 22% of patients were not receiving treatment at the time of the survey; 16% of these patients had never been prescribed treatment and symptom burden remained high, with over three quarters of patients symptomatic at survey date.

The current study also highlights the differences in treatment approach across types of PPF; for example, 7% of patients with polymyositis/dermatomyositis-ILD were not prescribed treatment at survey date compared with 35% of patients with uILD. This is comparable with another survey of physicians in Europe and the US, where 25–50% of patients with PPF did not receive drug treatment [38]. Despite the poor prognosis and high mortality associated with PPF, approximately 15% of patients had never received treatment for ILD at the time of the survey. An analysis of data from the Phase 3 trials of patients with IPF and other progressive fibrosing ILDs receiving either nintedanib or placebo, carried out over 52 weeks, found a similar disease course for both, underlining the possibility of a poor prognosis with PPF and the continued unmet need for timely diagnosis and treatment of the underlying ILD [34].

Only 25% of patients were in full-time employment at survey date, and 19% of those not in full-time employment were unable to work full-time due to their ILD (either working part-time, being retired, on long-term sick leave or unemployed). This aligns with research on the economic burden of PPF. In an economic model of PPF and SSc-ILD based on published data, the estimated annual aggregate income loss in the European Economic Area (accounting for annual sick days, early retirements, and permanently disabled patients) was €1,433 million and €220 million, respectively [39]. In addition, productivity loss due to job losses was €194 million and €26 million for PPF and SSc-ILD, respectively [39].

There are several limitations with the current study, including that the participating patients may not reflect the general population of patients with PPF with other underlying ILDs. The study population may also be skewed towards those more willing to consult physicians, or with more severe disease and undergoing monitoring for treatment response. The study was not based on a true random sample of physicians or patients, and no formal patient selection verification procedures were in place, with identification of the target patient group based on the judgement of the respondent physician. To minimise a potential selection bias and limit pre-selection by the physician, the sample comprised consecutive eligible patients, and all patients who met the eligibility criteria were included in the study. While the description of the type of patients included in this study reflects PPF, patients were not required to meet the 2022 ATS/ERS/JRS/ALAT guidelines [1] classification of PPF, as these criteria were not defined at the time of data collection. However, patients were required to have an ILD that the physician considered to be a progressive phenotype, and physicians did provide data on absolute decline in FVC and DLco and extent of disease on HRCT – factors used to define PPF according to guidelines [1]. While some patients may not fit the current guideline criteria despite a physician clinical classification that states their condition is progressing, this method is representative of physicians’ real-world classification of patients. It will be interesting to see how this evolves over time with the adoption of the ATS/ERS/JRS/ALAT guidelines for PPF. Multidisciplinary discussion (MDD) can facilitate a timely and accurate diagnosis of ILD, incorporating input from various clinicians, radiologists, pathologists and other specialists. Although the 2022 ATS/ERS/JRS/ALAT guidelines [1] focus on MDD for IPF, there is recognition of the importance of MDD for other ILDs as well [32, 33, 40, 41]. While the current study found that in just over 75% of cases no additional physicians were involved in the diagnosis, 83% of respondents reported being part of a multidisciplinary team, with most having a role in diagnosis and ongoing management. As diagnosis data were based on historical medical records, a single diagnosing physician may have been named, and the respondent may not have had access to further details regarding MDD of diagnosis.

## Conclusions

This real-world study adds to our understanding of patients with PPF currently under the care of physicians in Europe, finding that many patients experience misdiagnosis, delays in diagnosis and gaps in treatment, with some patients having never received treatment for ILD. These findings draw attention to the unmet needs around the ongoing patient burden in PPF, the importance of referring patients with fibrosing ILD to expert centres, regular monitoring for progression and the need for access to timely treatment. Further research is needed to improve clarity in the guidelines on the diagnosis, management and treatment of all types of fibrotic ILD with a progressive phenotype.

## Electronic supplementary material

Below is the link to the electronic supplementary material.

Supplementary Material 1

## Data Availability

The data that support the findings of this study are available from Adelphi Real World but restrictions apply to the availability of these data, which were used under license for the current study, and so are not publicly available. Data are, however, available from the authors upon reasonable request and with permission of Adelphi Real World.

## Abbreviations

ALAT: Latin American Thoracic Association
ATS: American Thoracic Society
COPD: Chronic obstructive pulmonary disease
CTD: Connective tissue disease
DLco: Diffusing capacity of the lungs for carbon monoxide
DSP: Disease Specific Programme
ERS: European Respiratory Society
FVC: Forced vital capacity
HCP: Healthcare professional
HCRU: Healthcare resource utilisation
HRCT: High-resolution computed tomography
ILD: Interstitial lung disease
iNSIP: Idiopathic non-specific interstitial pneumonia
IPF: Idiopathic pulmonary fibrosis
JRS: Japanese Respiratory Society
MDD: Multidisciplinary discussion
PPF: Progressive pulmonary fibrosis
PRF: Patient record form
RA: Rheumatoid arthritis
SD: Standard deviation
SSc: Systemic sclerosis
uILD: Unclassifiable ILD
