# Supplementary material for: Treatment patterns and patient journey in progressive pulmonary fibrosis: a cross-sectional survey
Source: Respir Res. 2024 Oct 9;25:364. doi: 10.1186/s12931-024-02995-9 (PMC11465623; doi:10.1186/s12931-024-02995-9)
Supplement: Supplementary file 1 — Supplementary Material 1 [file 12931_2024_2995_MOESM1_ESM.docx]

**Supplementary Materials**

**Treatment patterns and patient pathways in progressive pulmonary fibrosis: a cross-sectional survey**

Nazia Chaudhuri, Paolo Spagnolo, Claudia Valenzuela, Valeria C. Amatto, Oliver-Thomas Carter, Lauren Lee, Mark Small, Michael Kreuter

**Supplementary Methods**

*Sample size and confidence intervals*

As the objectives are descriptive, the sample size impacted the precision of estimates. Precision can be measured by the width of 95% confidence intervals (CIs). A larger sample would produce narrower CIs, indicating more precise estimates. The tables below show the width of 95% CIs for a categorical and a continuous variable for selected sample sizes (Table S1–2). For the categorical variable, a proportion of 50% was chosen as that produces the widest CI. For the continuous variable, a standardised version was chosen, where the standard deviation is 1.

**Table S1. Physician survey: Half-width/precision**

| **Type** | **95% CI parameter** | **n=40** | **n=50** | **n=53** | **n=61** | **n=265** |
| --- | --- | --- | --- | --- | --- | --- |
| Categorical^a^ | Max half-width (occurs around 50%) | ±16.2% | ±14.5% | ±14.9% | ±13.9% | ±6.4% |
| Continuous^b^ | Half-width (standardised) | ±0.320 | ±0.284 | ±0.276 | ±0.256 | ±0.121 |

^a^Using the Clopper-Pearson method.

^b^Assumes a normally distributed continuous variable and uses the t distribution.

CI, confidence interval.

**Table S2. Physician-reported patient data (PRF): Half-width/precision**

| **Type** | **95% CI parameter** | **n=184** | **n=271** | **n=276** | **n=328** | **n=1335** |
| --- | --- | --- | --- | --- | --- | --- |
| Categorical* | Max half-width (occurs around 50%) | ±7.4% | ±6.3% | ±6.1% | ±5.5% | ±2.8% |
| Continuous^†^ | Half-width (standardised) | ±0.145 | ±0.120 | ±0.118 | ±0.109 | ±0.054 |

*Using the Clopper-Pearson method.

^†^Assumes a normally distributed continuous variable and uses the t distribution.

CI, confidence interval.

**Supplementary Results**

**Figure S1. Physician-rated severity of ILD**

A. Physician-rated severity of ILD by country


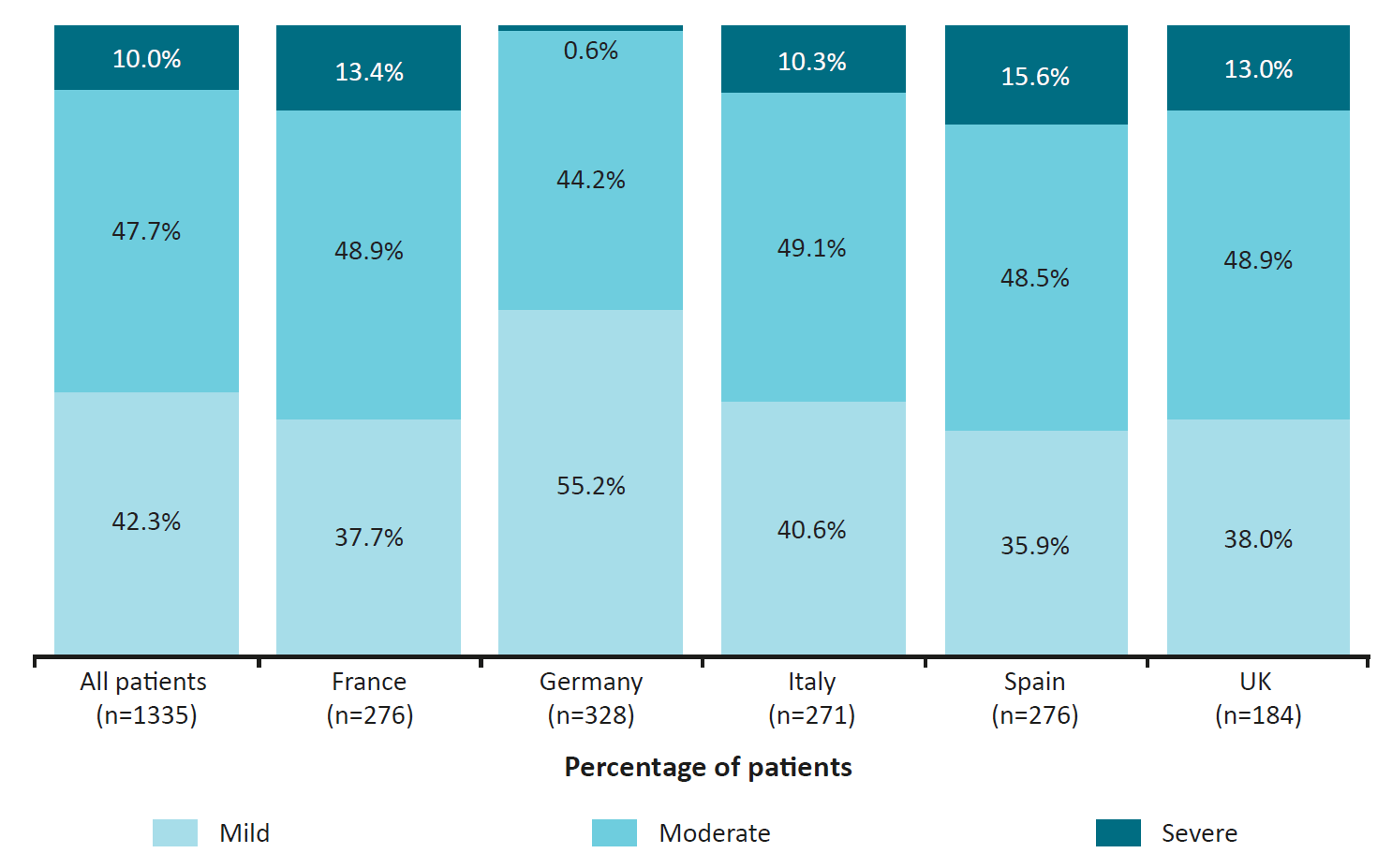


B. Physician-rated severity of ILD by physician speciality


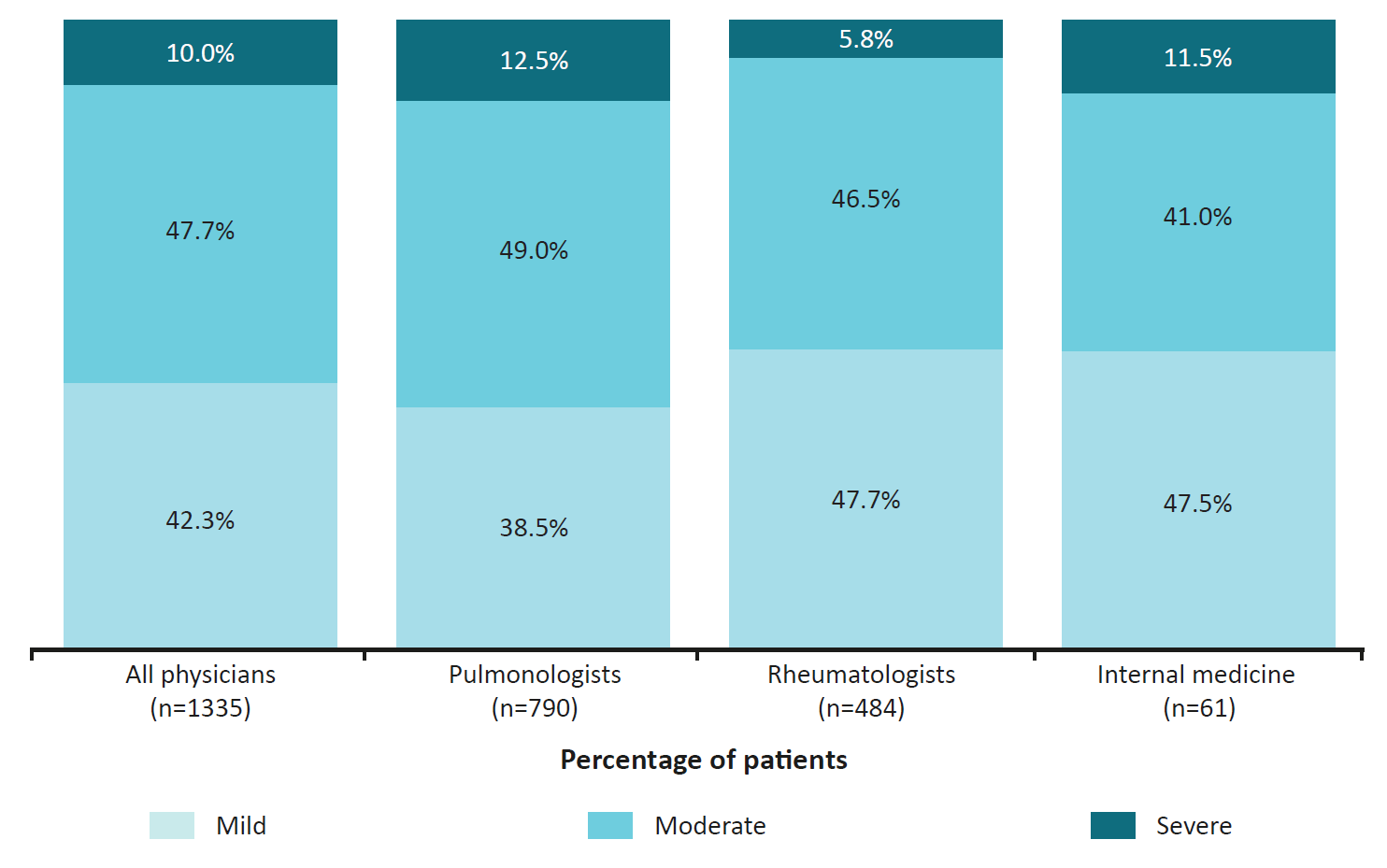


C. Physician-rated severity of ILD by type of ILD


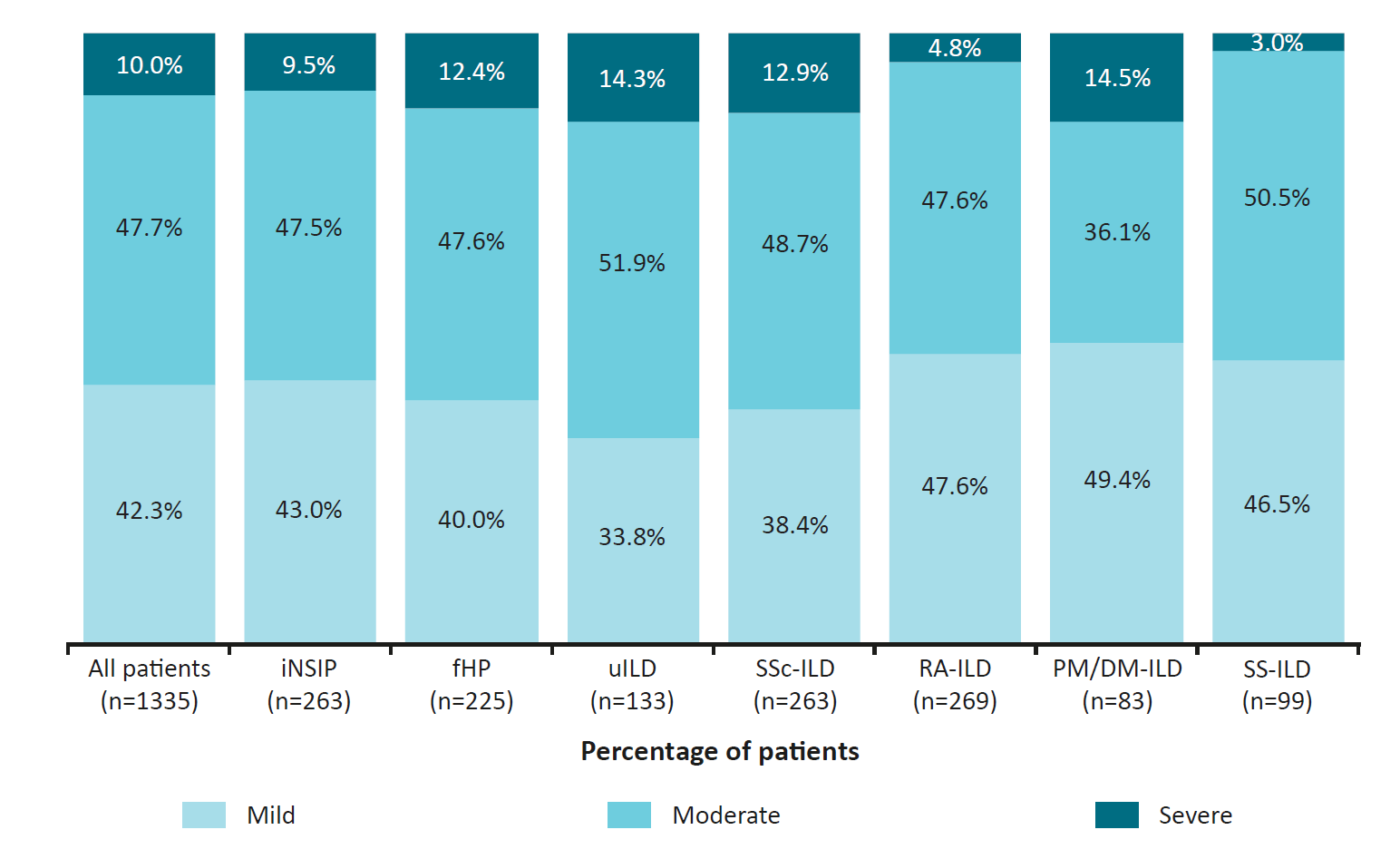


fHP, fibrotic hypersensitivity pneumonitis; ILD, interstitial lung disease; IM, internal medicine; iNSIP, idiopathic non-specific interstitial pneumonia; PM/DM-ILD, polymyositis- / dermatomyositis-associated ILD; PPF, progressive pulmonary fibrosis; RA‑ILD, rheumatoid arthritis-associated ILD; SSc-ILD, systemic sclerosis-associated ILD; SS‑ILD, Sjögren's-associated ILD; uILD, unclassifiable ILD; UK, United Kingdom.

**Figure S2. Physician-reported patient disease status over last 12 months according to time since diagnosis of ILD**
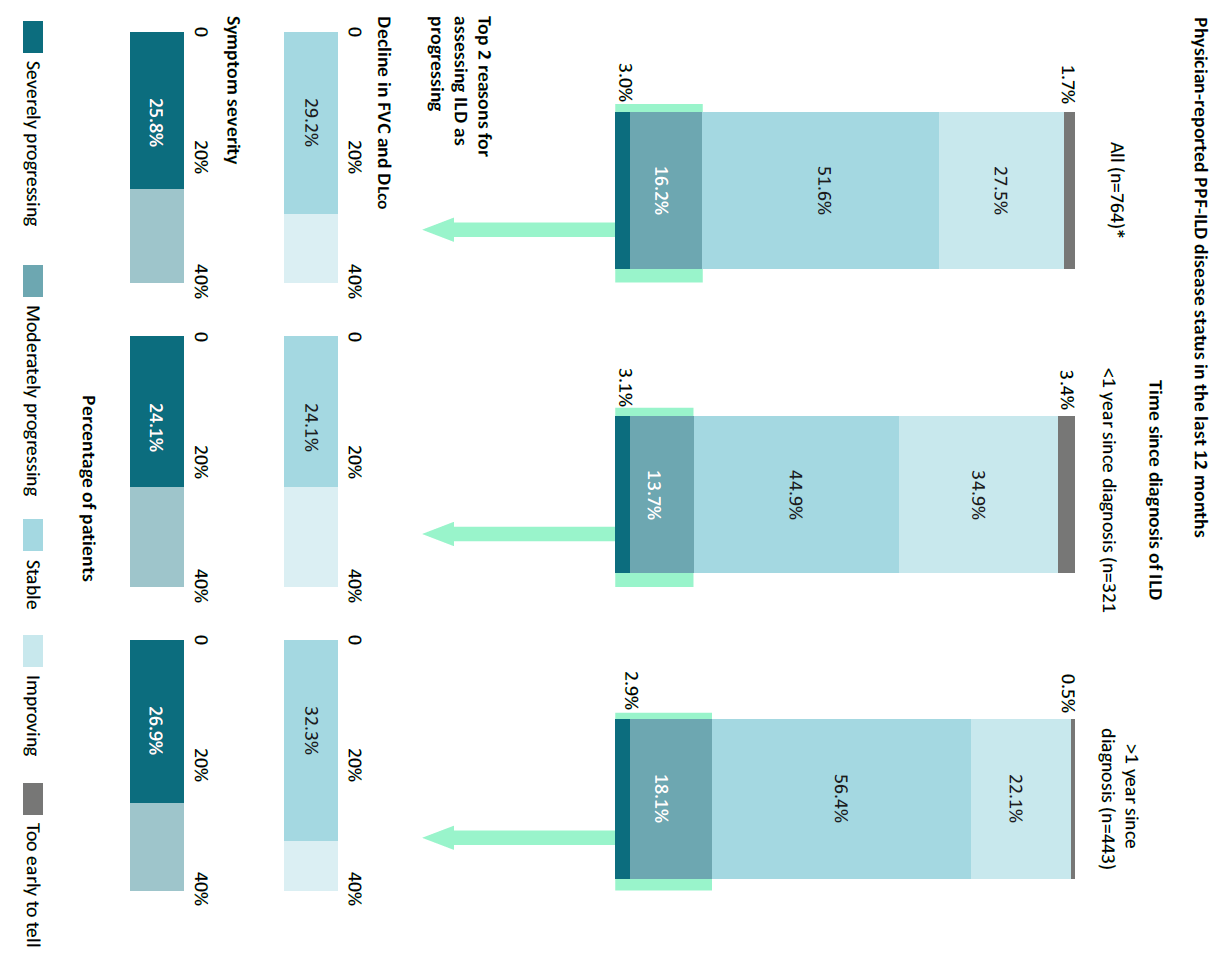


DLco, diffusing capacity of the lung for carbon monoxide; FVC, forced vital capacity; ILD, interstitial lung disease; PPF, progressive pulmonary fibrosis.

**Figure S3. Expected progression in the next 12 months according to lung function parameters**


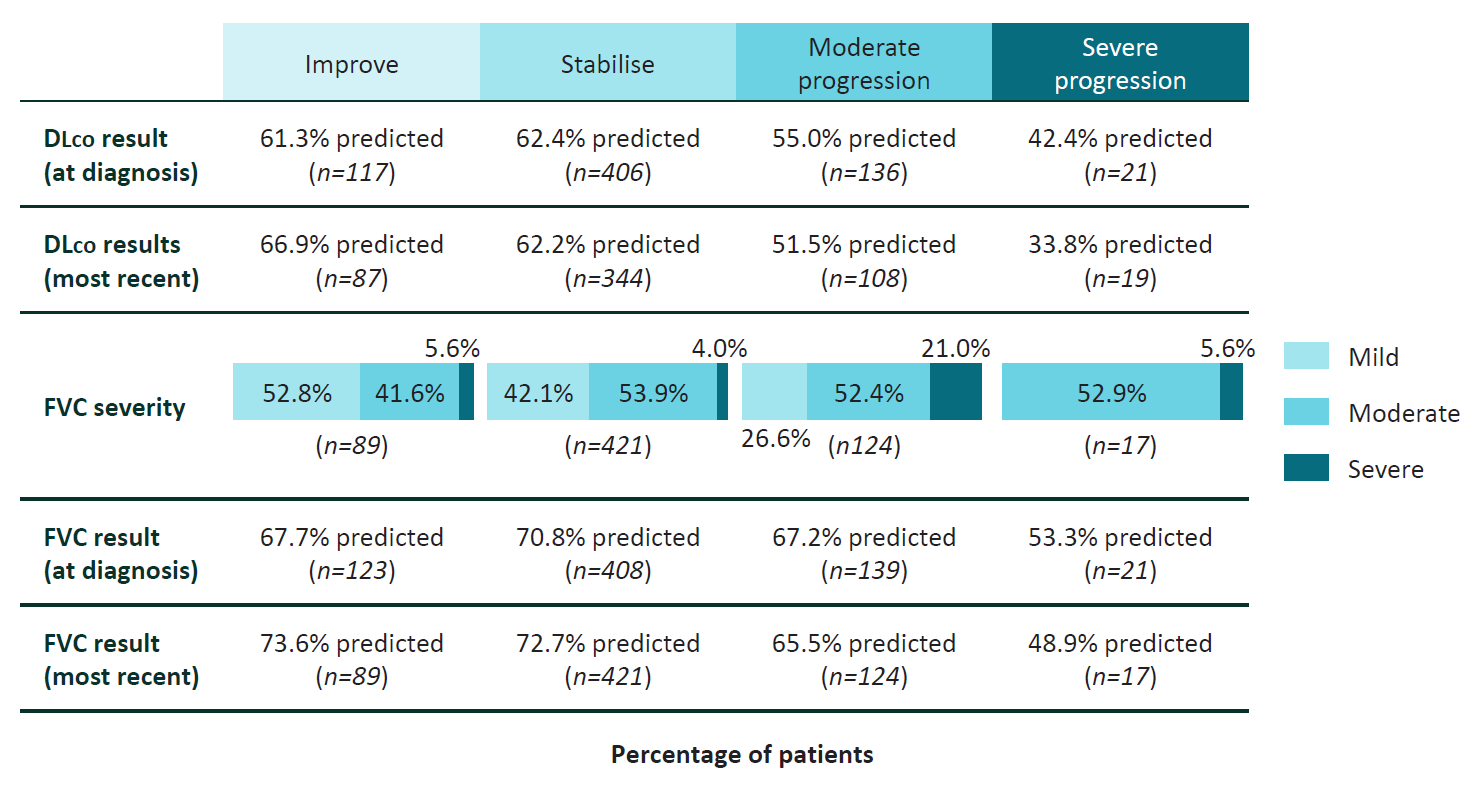


DLco, diffusing capacity of the lung for carbon monoxide; FVC, forced vital capacity; ILD, interstitial lung disease; PPF, progressive pulmonary fibrosis

**Figure S4. Next course of action if current treatment were inadequate by country**

**
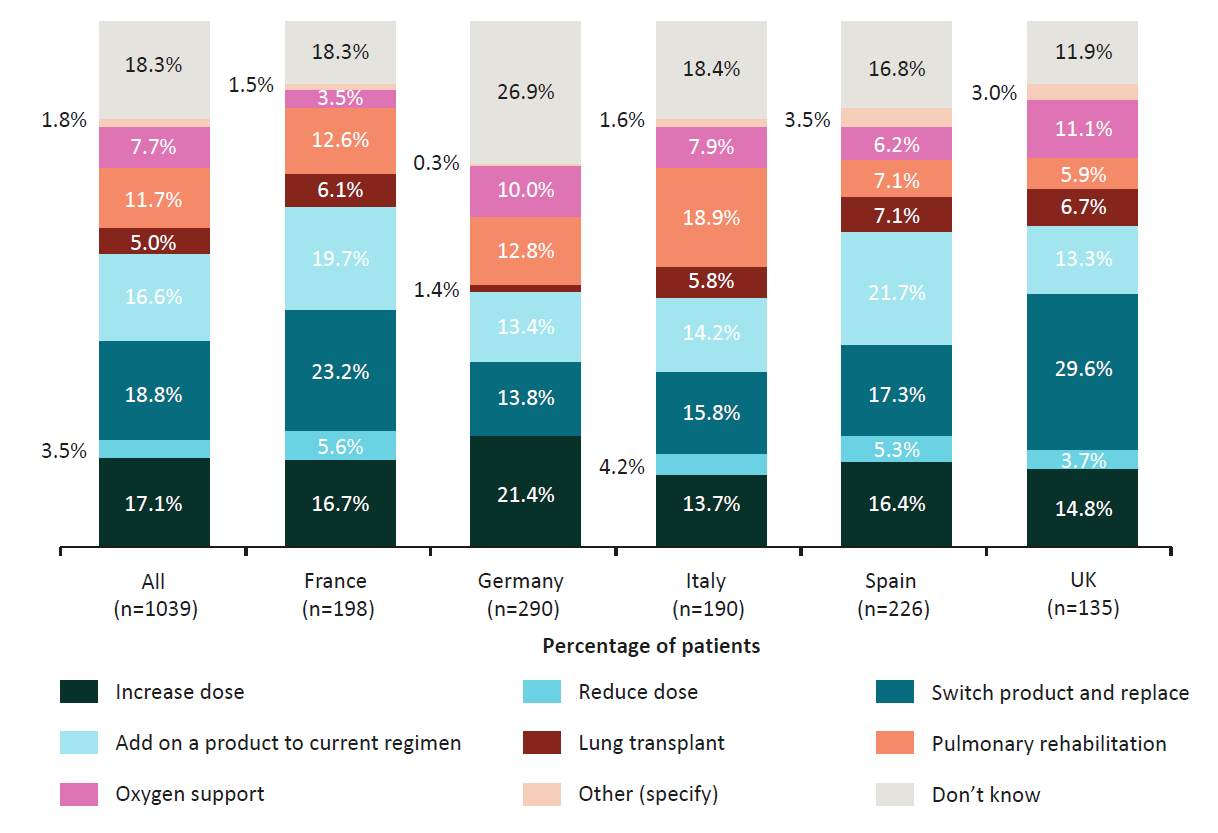
**

UK, United Kingdom.

**Table S3. PPF-ILD DSP study sample**

|  |  | **Physician specialty** | | |
| --- | --- | --- | --- | --- |
|  | **Total** | **Pulmonologist** | **Rheumatologist** | **Internal medicine** |
| Physician surveys | 265 | 175 | 82 | 8 |
| France | 40 | 22 | 10 | 8 |
| Germany | 61 | 41 | 20 | 0 |
| Italy | 61 | 41 | 20 | 0 |
| Spain | 53 | 37 | 16 | 0 |
| UK | 50 | 34 | 16 | 0 |
| Patient record forms | 1335 | 790 | 484 | 61 |
| France | 276 | 131 | 84 | 61 |
| Germany | 328 | 208 | 120 | 0 |
| Italy | 271 | 171 | 100 | 0 |
| Spain | 276 | 180 | 96 | 0 |
| UK | 184 | 100 | 84 | 0 |

DSP, Disease Specific Programme; PPF, progressive pulmonary fibrosis; UK, United Kingdom.

**Table S4. Patient demographics by country**

|  | **All patients** | **Country** | | | | |
| --- | --- | --- | --- | --- | --- | --- |
|  |  | **France** | **Germany** | **Italy** | **Spain** | **UK** |
| Physician-reported patient age, years | 1334 | 276 | 328 | 271 | 276 | 183 |
| Mean (SD) | 60.38 (11.59) | 60.89 (12.43) | 56.40 (9.99) | 61.70 (11.60) | 63.20 (11.35) | 60.56 (11.52) |
| BMI, kg/m^2^ | 1335 | 276 | 328 | 271 | 276 | 184 |
| Mean (SD) | 25.31 (3.89) | 24.64 (4.15) | 24.81 (3.36) | 24.96 (4.03) | 26.17 (3.36) | 26.39 (4.45) |
| Sex, n (%) | 1335 | 276 | 328 | 271 | 276 | 183 |
| Female | 776 (58.13) | 163 (59.06) | 177 (53.96) | 163 (60.15) | 160 (57.97) | 113 (61.75) |
| Patient ethnicity, n (%) | 1335 | 276 | 328 | 271 | 276 | 184 |
| White/Caucasian | 1217 (91.16) | 231 (83.70) | 314 (95.73) | 262 (96.68) | 259 (93.84) | 151 (82.07) |
| Asian (Indian subcontinent) | 11 (0.82) | 0 (0.00) | 1 (0.30) | 0 (0.00) | 0 (0.00) | 10 (5.43) |
| Asian (other) | 5 (0.37) | 1 (0.36) | 0 (0.00) | 0 (0.00) | 0 (0.00) | 4 (2.17) |
| Hispanic / Latino | 30 (2.25) | 3 (1.09) | 2 (0.61) | 8 (2.95) | 13 (4.71) | 4 (2.17) |
| Middle Eastern | 25 (1.87) | 18 (6.52) | 4 (1.22) | 0 (0.00) | 1 (0.36) | 2 (1.09) |
| Mixed race | 10 (0.75) | 1 (0.36) | 7 (2.13) | 0 (0.00) | 2 (0.72) | 0 (0.00) |
| Afro-Caribbean | 28 (2.10) | 20 (7.25) | 0 (0.00) | 1 (0.37) | 0 (0.00) | 7 (3.80) |
| Southeast Asian | 9 (0.67) | 2 (0.72) | 0 (0.00) | 0 (0.00) | 1 (0.36) | 6 (3.26) |
| Other | 0 (0.00) | 0 (0.00) | 0 (0.00) | 0 (0.00) | 0 (0.00) | 0 (0.00) |
| Patient smoking status, n (%) | 1335 | 276 | 328 | 271 | 276 | 184 |
| Current smoker | 98 (7.34) | 28 (10.14) | 19 (5.79) | 21 (7.75) | 20 (7.25) | 10 (5.43) |
| Ex-smoker | 612 (45.84) | 104 (37.68) | 201 (61.28) | 120 (44.28) | 108 (39.13) | 79 (42.93) |
| Never smoked | 588 (44.04) | 143 (51.81) | 100 (30.49) | 110 (40.59) | 146 (52.90) | 89 (48.37) |
| Don't know | 37 (2.77) | 1 (0.36) | 8 (2.44) | 20 (7.38) | 2 (0.72) | 6 (3.26) |
| Patient employment status, n (%) | 1335 | 276 | 328 | 271 | 276 | 184 |
| Working full time | 336 (25.17) | 71 (25.72) | 124 (37.80) | 62 (22.88) | 54 (19.57) | 25 (13.59) |
| Working part time | 145 (10.86) | 17 (6.16) | 31 (9.45) | 43 (15.87) | 21 (7.61) | 33 (17.93) |
| On long-term sick leave | 76 (5.69) | 19 (6.88) | 15 (4.57) | 4 (1.48) | 21 (7.61) | 17 (9.24) |
| Homemaker | 161 (12.06) | 11 (3.99) | 53 (16.16) | 29 (10.70) | 51 (18.48) | 17 (9.24) |
| Student | 1 (0.07) | 0 (0.00) | 0 (0.00) | 0 (0.00) | 1 (0.36) | 0 (0.00) |
| Retired | 517 (38.73) | 137 (49.64) | 91 (27.74) | 102 (37.64) | 110 (39.86) | 77 (41.85) |
| Unemployed | 68 (5.09) | 21 (7.61) | 14 (4.27) | 11 (4.06) | 14 (5.07) | 8 (4.35) |
| Don’t know | 31 (2.32) | 0 (0.00) | 0 (0.00) | 20 (7.38) | 4 (1.45) | 7 (3.80) |

SD, standard deviation; UK, United Kingdom.

**Table S5. Patient demographics by physician specialty**

|  | **All patients** | **Physician speciality** | | |
| --- | --- | --- | --- | --- |
|  |  | **Pulmonologist** | **Rheumatologist** | **Internal medicine** |
| Physician-reported patient age, years | 1334 | 789 | 484 | 61 |
| Mean (SD) | 60.38 (11.59) | 62.63 (11.42) | 58.03 (10.69) | 49.98 (11.36) |
| BMI, kg/m^2^ | 1335 | 790 | 484 | 61 |
| Mean (SD) | 25.31 (3.89) | 25.73 (3.89) | 24.86 (3.79) | 23.37 (3.71) |
| Sex, n (%) | 1335 | 790 | 484 | 61 |
| Female | 776 (58.13) | 390 (49.43) | 342 (70.66) | 44 (72.13) |
| Patient ethnicity, n (%) | 1335 | 790 | 484 | 61 |
| White/Caucasian | 1217 (91.16) | 735 (93.04) | 436 (90.08) | 46 (75.41) |
| Asian (Indian subcontinent) | 11 (0.82) | 2 (0.25) | 9 (1.86) | 0 (0.00) |
| Asian (other) | 5 (0.37) | 4 (0.51) | 1 (0.21) | 0 (0.00) |
| Hispanic / Latino | 30 (2.25) | 16 (2.03) | 14 (2.89) | 0 (0.00) |
| Middle Eastern | 25 (1.87) | 11 (1.39) | 9 (1.86) | 5 (8.20) |
| Mixed race | 10 (0.75) | 9 (1.14) | 0 (0.00) | 1 (1.64) |
| Afro-Caribbean | 28 (2.10) | 9 (1.14) | 11 (2.27) | 8 (13.11) |
| Southeast Asian | 9 (0.67) | 4 (0.51) | 4 (0.83) | 1 (1.64) |
| Other | 0 (0.00) | 0 (0.00) | 0 (0.00) | 0 (0.00) |
| Patient smoking status, n (%) | 1335 | 790 | 484 | 61 |
| Current smoker | 98 (7.34) | 64 (8.10) | 24 (4.96) | 10 (16.39) |
| Ex-smoker | 612 (45.84) | 360 (45.57) | 239 (49.38) | 13 (21.31) |
| Never smoked | 588 (44.04) | 348 (44.05) | 202 (41.74) | 38 (62.30) |
| Don't know | 37 (2.77) | 18 (2.28) | 19 (3.93) | 0 (0.00) |
| Patient employment status, n (%) | 1335 | 790 | 484 | 61 |
| Working full time | 336 (25.17) | 183 (23.16) | 127 (26.24) | 26 (42.62) |
| Working part time | 145 (10.86) | 60 (7.59) | 80 (16.53) | 5 (8.20) |
| On long-term sick leave | 76 (5.69) | 46 (5.82) | 22 (4.55) | 8 (13.11) |
| Homemaker | 161 (12.06) | 89 (11.27) | 68 (14.05) | 4 (6.56) |
| Student | 1 (0.07) | 0 (0.00) | 1 (0.21) | 0 (0.00) |
| Retired | 517 (38.73) | 362 (45.82) | 145 (29.96) | 10 (16.39) |
| Unemployed | 68 (5.09) | 31 (3.92) | 29 (5.99) | 8 (13.11) |
| Don’t know | 31 (2.32) | 19 (2.41) | 12 (2.48) | 0 (0.00) |

SD, standard deviation.

**Table S6. Patient demographics by type of ILD**

|  | **All patients** | **Type of ILD** | | | | | | |
| --- | --- | --- | --- | --- | --- | --- | --- | --- |
|  |  | **iNSIP** | **fHP** | **uILD** | **SSc-ILD** | **RA-ILD** | **PM/DM-ILD** | **SS-ILD** |
| Physician-reported patient age, years | 1334 | 262 | 225 | 133 | 263 | 269 | 83 | 99 |
| Mean (SD) | 60.38 (11.59) | 62.50 (11.09) | 62.08 (11.68) | 66.41 (12.02) | 56.14 (10.56) | 61.06 (10.56) | 53.83 (11.68) | 57.76 (10.73) |
| BMI, kg/m^2^ | 1335 | 263 | 225 | 133 | 263 | 269 | 83 | 99 |
| Mean (SD) | 25.31 (3.89) | 25.53 (3.24) | 26.29 (4.00) | 25.88 (4.20) | 24.07 (3.99) | 25.42 (4.08) | 25.41 (3.57) | 24.58 (3.39) |
| Sex, n (%) | 1335 | 263 | 225 | 133 | 263 | 269 | 83 | 99 |
| Female | 776 (58.13) | 126 (47.90) | 68 (30.22) | 56 (42.10) | 223 (84.79) | 166 (61.71) | 53 (63.86) | 84 (84.84) |
| Patient ethnicity, n (%) | 1335 | 263 | 225 | 133 | 263 | 269 | 83 | 99 |
| White/Caucasian | 1217 (91.16) | 245 (93.16) | 216 (96.00) | 121 (90.98) | 237 (90.11) | 251 (93.31) | 61 (73.49) | 86 (86.87) |
| Asian (Indian subcontinent) | 11 (0.82) | 0 (0.00) | 1 (0.44) | 1 (0.75) | 4 (1.52) | 0 (0.00) | 2 (2.41) | 3 (3.03) |
| Asian (other) | 5 (0.37) | 0 (0.00) | 0 (0.00) | 1 (0.75) | 2 (0.76) | 0 (0.00) | 2 (2.41) | 0 (0.00) |
| Hispanic / Latino | 30 (2.25) | 5 (1.90) | 3 (1.33) | 3 (2.26) | 6 (2.28) | 6 (2.23) | 5 (6.02) | 2 (2.02) |
| Middle Eastern | 25 (1.87) | 1 (0.38) | 1 (0.44) | 4 (3.01) | 7 (2.66) | 5 (1.86) | 5 (6.02) | 2 (2.02) |
| Mixed race | 10 (0.75) | 8 (3.04) | 0 (0.00) | 0 (0.00) | 0 (0.00) | 1 (0.37) | 0 (0.00) | 1 (1.01) |
| Afro-Caribbean | 28 (2.10) | 3 (1.14) | 3 (1.33) | 2 (1.50) | 5 (1.90) | 3 (1.12) | 7 (8.43) | 5 (5.05) |
| Southeast Asian | 9 (0.67) | 1 (0.38) | 1 (0.44) | 1 (0.75) | 2 (0.76) | 3 (1.12) | 1 (1.20) | 0 (0.00) |
| Other | 0 (0.00) | 0 (0.00) | 0 (0.00) | 0 (0.00) | 0 (0.00) | 0 (0.00) | 0 (0.00) | 0 (0.00) |
| Patient smoking  status, n (%) | 1335 | 263 | 225 | 133 | 263 | 269 | 83 | 99 |
| Current smoker | 98 (7.34) | 25 (9.51) | 19 (8.44) | 12 (9.02) | 11 (4.18) | 20 (7.43) | 5 (6.02) | 6 (6.06) |
| Ex-smoker | 612 (45.84) | 128 (48.67) | 104 (46.22) | 59 (44.36) | 107 (40.68) | 140 (52.04) | 32 (38.55) | 42 (42.42) |
| Never smoked | 588 (44.04) | 103 (39.16) | 99 (44.00) | 60 (45.11) | 132 (50.19) | 99 (36.80) | 46 (55.42) | 49 (49.49) |
| Don't know | 37 (2.77) | 7 (2.66) | 3 (1.33) | 2 (1.50) | 13 (4.94) | 10 (3.72) | 0 (0.00) | 2 (2.02) |
| Patient employment status, n (%) | 1335 | 263 | 225 | 133 | 263 | 269 | 83 | 99 |
| Working full time | 336 (25.17) | 70 (26.62) | 64 (28.44) | 20 (15.04) | 60 (22.81) | 65 (24.16) | 28 (33.73) | 29 (29.29) |
| Working part time | 145 (10.86) | 20 (7.60) | 19 (8.44) | 6 (4.51) | 42 (15.97) | 27 (10.04) | 14 (16.87) | 17 (17.17) |
| On long-term sick leave | 76 (5.69) | 10 (3.80) | 14 (6.22) | 7 (5.26) | 24 (9.13) | 13 (4.83) | 7 (8.43) | 1 (1.01) |
| Homemaker | 161 (12.06) | 37 (14.07) | 15 (6.67) | 11 (8.27) | 49 (18.63) | 29 (10.78) | 5 (6.02) | 15 (15.15) |
| Student | 1 (0.07) | 0 (0.00) | 0 (0.00) | 0 (0.00) | 0 (0.00) | 0 (0.00) | 1 (1.20) | 0 (0.00) |
| Retired | 517 (38.73) | 114 (43.35) | 98 (43.56) | 79 (59.40) | 64 (24.33) | 116 (43.12) | 17 (20.48) | 29 (29.29) |
| Unemployed | 68 (5.09) | 7 (2.66) | 6 (2.67) | 10 (7.52) | 15 (5.70) | 13 (4.83) | 10 (12.05) | 7 (7.07) |
| Don’t know | 31 (2.32) | 5 (1.90) | 9 (4.00) | 0 (0.00) | 9 (3.42) | 6 (2.23) | 1 (1.20) | 1 (1.01) |

fHP, fibrotic hypersensitivity pneumonitis; ILD, interstitial lung disease; iNSIP, idiopathic non-specific interstitial pneumonia; PM/DM-ILD, polymyositis- / dermatomyositis-associated ILD; RA‑ILD, rheumatoid arthritis-associated ILD; SD, standard deviation; SSc-ILD, systemic sclerosis-associated ILD; SS‑ILD, Sjögren's-associated ILD; uILD, unclassifiable ILD.

**Table S7.** **Physician-reported reasons for disease progression by country**

|  | **All patients**  **(n=260)** | **County** | | | | |
| --- | --- | --- | --- | --- | --- | --- |
|  |  | **France**  **(n=58)** | **Germany**  **(n=32)** | **Italy**  **(n=65)** | **Spain**  **(n=60)** | **UK**  **(n=45)** |
| Reasons for progressing, n (%) |  |  |  |  |  |  |
| Acute exacerbation of ILD | 13 (5.00) | 4 (6.90) | 0 (0.00) | 4 (6.15) | 3 (5.00) | 2 (4.44) |
| Symptom severity | 71 (27.31) | 13 (22.41) | 11 (34.38) | 19 (29.23) | 9 (15.00) | 19 (42.22) |
| Symptom frequency | 20 (7.69) | 6 (10.34) | 5 (15.62) | 6 (9.23) | 2 (3.33) | 1 (2.22) |
| Decline in FVC only | 19 (7.31) | 7 (12.07) | 3 (9.38) | 2 (3.08) | 5 (8.33) | 2 (4.44) |
| Decline in DLco only | 14 (5.38) | 0 (0.00) | 2 (6.25) | 2 (3.08) | 6 (10.00) | 4 (8.89) |
| Decline in FVC and DLco | 67 (25.77) | 13 (22.41) | 5 (15.62) | 18 (27.69) | 24 (40.00) | 7 (15.56) |
| Requires lung transplant | 8 (3.08) | 4 (6.90) | 0 (0.00) | 1 (1.54) | 2 (3.33) | 1 (2.22) |
| Change in underlying disease severity | 2 (0.77) | 0 (0.00) | 0 (0.00) | 0 (0.00) | 0 (0.00) | 2 (4.44) |
| Initiation of new treatment to treat their ILD | 5 (1.92) | 1 (1.72) | 0 (0.00) | 1 (1.54) | 1 (1.67) | 2 (4.44) |
| Change in the dose / frequency of their current treatment | 0 (0.00) | 0 (0.00) | 0 (0.00) | 0 (0.00) | 0 (0.00) | 0 (0.00) |
| Increased fibrosis of the lungs as demonstrated by CT / HRCT scan | 37 (14.23) | 9 (15.52) | 6 (18.75) | 11 (16.92) | 6 (10.00) | 5 (11.11) |
| Other | 3 (1.15) | 1 (1.72) | 0 (0.00) | 1 (1.54) | 1 (1.67) | 0 (0.00) |
| None | 1 (0.38) | 0 (0.00) | 0 (0.00) | 0 (0.00) | 1 (1.67) | 0 (0.00) |

CT, computed tomography; DLco, diffusing capacity of the lung for carbon monoxide; FVC, forced vital capacity; HRCT, high-resolution CT; ILD, interstitial lung disease; PPF, progressive pulmonary fibrosis; UK, United Kingdom.

**Table S8.** **Physician-reported reasons for disease progression by physician specialty**

|  | **All patients**  **(n=260)** | **Physician speciality** | | |
| --- | --- | --- | --- | --- |
|  |  | **Pulmonologist**  **(n=208)** | **Rheumatologist**  **(n=46)** | **Internal medicine**  **(n=6)** |
| Reasons for progressing, n (%) | 260 | 208 | 46 | 6 |
| Acute exacerbation of ILD | 13 (5.00) | 9 (4.33) | 4 (8.70) | 0 (0.00) |
| Symptom severity | 71 (27.31) | 55 (26.44) | 15 (32.61) | 1 (16.67) |
| Symptom frequency | 20 (7.69) | 17 (8.17) | 3 (6.52) | 0 (0.00) |
| Decline in FVC only | 19 (7.31) | 15 (7.21) | 3 (6.52) | 1 (16.67) |
| Decline in DLco only | 14 (5.38) | 11 (5.29) | 3 (6.52) | 0 (0.00) |
| Decline in FVC and DLco | 67 (25.77) | 57 (27.40) | 8 (17.39) | 2 (33.33) |
| Requires lung transplant | 8 (3.08) | 5 (2.40) | 1 (2.17) | 2 (33.33) |
| Change in underlying disease severity | 2 (0.77) | 1 (0.48) | 1 (2.17) | 0 (0.00) |
| Initiation of new treatment to treat their ILD | 5 (1.92) | 4 (1.92) | 1 (2.17) | 0 (0.00) |
| Change in the dose / frequency of their current treatment | 0 (0.00) | 0 (0.00) | 0 (0.00) | 0 (0.00) |
| Increased fibrosis of the lungs as demonstrated by CT / HRCT scan | 37 (14.23) | 32 (15.38) | 5 (10.87) | 0 (0.00) |
| Other | 3 (1.15) | 1 (0.48) | 2 (4.35) | 0 (0.00) |
| None | 1 (0.38) | 1 (0.48) | 0 (0.00) | 0 (0.00) |

CT, computed tomography; DLco, diffusing capacity of the lung for carbon monoxide; FVC, forced vital capacity; HRCT, high-resolution CT; ILD, interstitial lung disease; PPF, progressive pulmonary fibrosis.

**Table S9.** **Physician-reported reasons for disease progression by type of ILD**

|  | **All patients**  **(n=260)** | **Type of ILD** | | | | | | |
| --- | --- | --- | --- | --- | --- | --- | --- | --- |
|  |  | **iNSIP**  **(n=66)** | **fHP**  **(n=44)** | **uILD**  **(n=46)** | **SSc-ILD**  **(n=55)** | **RA-ILD**  **(n=24)** | **PM/DM-ILD**  **(n=12)** | **SS-ILD**  **(n=13)** |
| Reasons for progressing, n (%) | 260 | 66 | 44 | 46 | 55 | 24 | 12 | 13 |
| Acute exacerbation of ILD | 13 (5.00) | 4 (6.06) | 0 (0.00) | 2 (4.35) | 0 (0.00) | 5 (20.83) | 2 (16.67) | 0 (0.00) |
| Symptom severity | 71 (27.31) | 17 (25.76) | 13 (29.55) | 14 (30.43) | 16 (29.09) | 6 (25.00) | 2 (16.67) | 3 (23.08) |
| Symptom frequency | 20 (7.69) | 6 (9.09) | 3 (6.82) | 2 (4.35) | 5 (9.09) | 2 (8.33) | 1 (8.33) | 1 (7.69) |
| Decline in FVC only | 19 (7.31) | 6 (9.09) | 1 (2.27) | 6 (13.04) | 2 (3.64) | 2 (8.33) | 1 (8.33) | 1 (7.69) |
| Decline in DLco only | 14 (5.38) | 6 (9.09) | 0 (0.00) | 3 (6.52) | 4 (7.27) | 1 (4.17) | 0 (0.00) | 0 (0.00) |
| Decline in FVC and DLco | 67 (25.77) | 17 (25.76) | 15 (34.09) | 7 (15.22) | 16 (29.09) | 7 (29.17) | 2 (16.67) | 3 (23.08) |
| Requires lung transplant | 8 (3.08) | 0 (0.00) | 2 (4.55) | 2 (4.35) | 2 (3.64) | 1 (4.17) | 1 (8.33) | 0 (0.00) |
| Change in underlying disease severity | 2 (0.77) | 0 (0.00) | 0 (0.00) | 0 (0.00) | 2 (3.64) | 0 (0.00) | 0 (0.00) | 0 (0.00) |
| Initiation of new treatment to treat their ILD | 5 (1.92) | 2 (3.03) | 1 (2.27) | 0 (0.00) | 1 (1.82) | 0 (0.00) | 0 (0.00) | 1 (7.69) |
| Change in the dose / frequency of their current treatment | 0 (0.00) | 0 (0.00) | 0 (0.00) | 0 (0.00) | 0 (0.00) | 0 (0.00) | 0 (0.00) | 0 (0.00) |
| Increased fibrosis of the lungs as demonstrated by CT / HRCT scan | 37 (14.23) | 7 (10.61) | 9 (20.45) | 9 (19.57) | 5 (9.09) | 0 (0.00) | 3 (25.00) | 4 (30.77) |
| Other | 3 (1.15) | 0 (0.00) | 0 (0.00) | 1 (2.17) | 2 (3.64) | 0 (0.00) | 0 (0.00) | 0 (0.00) |
| None | 1 (0.38) | 1 (1.52) | 0 (0.00) | 0 (0.00) | 0 (0.00) | 0 (0.00) | 0 (0.00) | 0 (0.00) |

CT, computed tomography; DLco, diffusing capacity of the lung for carbon monoxide; fHP, fibrotic hypersensitivity pneumonitis; FVC, forced vital capacity; HRCT, high-resolution CT; ILD, interstitial lung disease; iNSIP, idiopathic non-specific interstitial pneumonia; PM/DM-ILD, polymyositis- / dermatomyositis-associated ILD; RA‑ILD, rheumatoid arthritis-associated ILD; SSc-ILD, systemic sclerosis-associated ILD; SS‑ILD, Sjögren's-associated ILD; uILD, unclassifiable ILD.

**Table S10. Most frequent comorbidities reported at survey date by country**

|  |  | **Country** | | | | |
| --- | --- | --- | --- | --- | --- | --- |
|  | **All patients**  **(n=1335)** | **France**  **(n=276)** | **Germany**  **(n=328)** | **Italy**  **(n=271)** | **Spain**  **(n=276)** | **UK**  **(n=184)** |
| Comorbidities, n (%) |  |  |  |  |  |  |
| Anxiety | 226 (16.93) | 62 (22.46) | 10 (3.05) | 67 (24.72) | 53 (19.20) | 34 (18.48) |
| Gastroesophageal reflux | 207 (15.51) | 37 (13.41) | 16 (4.88) | 83 (30.63) | 42 (15.22) | 29 (15.76) |
| Depression | 179 (13.41) | 33 (11.96) | 22 (6.71) | 41 (15.13) | 50 (18.12) | 33 (17.93) |
| Diabetes without chronic complications | 134 (10.04) | 24 (8.70) | 42 (12.80) | 24 (8.86) | 32 (11.59) | 12 (6.52) |
| Pulmonary hypertension | 120 (8.99) | 21 (7.61) | 16 (4.88) | 34 (12.55) | 29 (10.51) | 20 (10.87) |
| Chronic obstructive pulmonary disease | 102 (7.64) | 26 (9.42) | 22 (6.71) | 27 (9.96) | 13 (4.71) | 14 (7.61) |
| Myocardial infarction | 84 (6.29) | 25 (9.06) | 9 (2.74) | 25 (9.23) | 13 (4.71) | 12 (6.52) |
| Coronary artery disease | 79 (5.92) | 20 (7.25) | 14 (4.27) | 15 (5.54) | 11 (3.99) | 19 (10.33) |
| Peripheral vascular disease | 75 (5.62) | 15 (5.43) | 10 (3.05) | 25 (9.23) | 12 (4.35) | 13 (7.07) |
| Obstructive sleep apnoea / apnoea | 68 (5.09) | 20 (7.25) | 9 (2.74) | 9 (3.32) | 23 (8.33) | 7 (3.80) |

UK, United Kingdom.

**Table S11. Most frequent comorbidities reported at survey date by physician specialty**

|  |  | **Physician specialty** | | |
| --- | --- | --- | --- | --- |
|  | **All patients**  **(n=1335)** | **Pulmonologist**  **(n=790)** | **Rheumatologist**  **(n=484)** | **Internal medicine**  **(n=61)** |
| Comorbidities, n (%) |  |  |  |  |
| Anxiety | 226 (16.93) | 134 (16.96) | 79 (16.32) | 13 (21.31) |
| Gastroesophageal reflux | 207 (15.51) | 134 (16.96) | 62 (12.81) | 11 (18.03) |
| Depression | 179 (13.41) | 98 (12.41) | 73 (15.08) | 8 (13.11) |
| Diabetes without chronic complications | 134 (10.04) | 93 (11.77) | 35 (7.23) | 6 (9.84) |
| Pulmonary hypertension | 120 (8.99) | 77 (9.75) | 42 (8.68) | 1 (1.64) |
| Chronic obstructive pulmonary disease | 102 (7.64) | 79 (10.00) | 18 (3.72) | 5 (8.20) |
| Myocardial infarction | 84 (6.29) | 67 (8.48) | 14 (2.89) | 3 (4.92) |
| Coronary artery disease | 79 (5.92) | 60 (7.59) | 19 (3.93) | 0 (0.00) |
| Peripheral vascular disease | 75 (5.62) | 52 (6.58) | 23 (4.75) | 0 (0.00) |
| Obstructive sleep apnoea / apnoea | 68 (5.09) | 58 (7.34) | 7 (1.45) | 3 (4.92) |

**Table S12. Most frequent comorbidities reported at survey date by type of ILD**

|  |  | **Type of ILD** | | | | | | |
| --- | --- | --- | --- | --- | --- | --- | --- | --- |
|  | **All patients**  **(n=1335)** | **iNSIP**  **(n=263)** | **fHP**  **(n=225)** | **uILD**  **(n=133)** | **SSc-ILD**  **(n=263)** | **RA-ILD**  **(n=269)** | **PM/DM-ILD**  **(n=83)** | **SS-ILD**  **(n=99)** |
| Comorbidities, n (%) |  |  |  |  |  |  |  |  |
| Anxiety | 226 (16.93) | 38 (14.45) | 35 (15.56) | 21 (15.79) | 59 (22.43) | 42 (15.61) | 15 (18.07) | 16 (16.16) |
| Gastroesophageal reflux | 207 (15.51) | 38 (14.45) | 32 (14.22) | 23 (17.29) | 70 (26.62) | 24 (8.92) | 8 (9.64) | 12 (12.12) |
| Depression | 179 (13.41) | 31 (11.79) | 27 (12.00) | 20 (15.04) | 42 (15.97) | 32 (11.90) | 13 (15.66) | 14 (14.14) |
| Diabetes without chronic complications | 134 (10.04) | 40 (15.21) | 24 (10.67) | 17 (12.78) | 20 (7.60) | 21 (7.81) | 3 (3.61) | 9 (9.09) |
| Pulmonary hypertension | 120 (8.99) | 23 (8.75) | 16 (7.11) | 15 (11.28) | 38 (14.45) | 16 (5.95) | 5 (6.02) | 7 (7.07) |
| Chronic obstructive pulmonary disease | 102 (7.64) | 25 (9.51) | 22 (9.78) | 21 (15.79) | 8 (3.04) | 19 (7.06) | 1 (1.20) | 6 (6.06) |
| Myocardial infarction | 84 (6.29) | 24 (9.13) | 20 (8.89) | 21 (15.79) | 4 (1.52) | 13 (4.83) | 2 (2.41) | 0 (0.00) |
| Coronary artery disease | 79 (5.92) | 21 (7.98) | 22 (9.78) | 11 (8.27) | 5 (1.90) | 19 (7.06) | 0 (0.00) | 1 (1.01) |
| Peripheral vascular disease | 75 (5.62) | 11 (4.18) | 19 (8.44) | 12 (9.02) | 18 (6.84) | 10 (3.72) | 3 (3.61) | 2 (2.02) |
| Obstructive sleep apnoea / apnoea | 68 (5.09) | 15 (5.70) | 23 (10.22) | 17 (12.78) | 2 (0.76) | 6 (2.23) | 2 (2.41) | 3 (3.03) |

fHP, fibrotic hypersensitivity pneumonitis; ILD, interstitial lung disease; iNSIP, idiopathic non-specific interstitial pneumonia; PM/DM-ILD, polymyositis- / dermatomyositis-associated ILD; RA‑ILD, rheumatoid arthritis-associated ILD; SSc-ILD, systemic sclerosis-associated ILD; SS‑ILD, Sjögren's-associated ILD; uILD, unclassifiable ILD.

**Table S13. Patients currently receiving treatment and reasons for never prescribing treatment by country**

|  | **All patients** | **Country** | | | | |
| --- | --- | --- | --- | --- | --- | --- |
|  |  | **France** | **Germany** | **Italy** | **Spain** | **UK** |
| Patients currently prescribed a treatment for their ILD, n (%) | 1335 | 276 | 328 | 271 | 276 | 184 |
| Yes | 1039 (77.8) | 198 (71.7) | 290 (88.4) | 190 (70.1) | 226 (81.9) | 135 (73.4) |
| No, but previously prescribed treatment | 88 (6.6) | 16 (5.8) | 7 (2.1) | 24 (8.9) | 19 (6.9) | 22 (12.0) |
| No, has never been prescribed treatment | 208 (15.6) | 62 (22.5) | 31 (9.5) | 57 (21.0) | 31 (11.2) | 27 (14.7) |
| Reason for never prescribing treatment for ILD, n (%) | 208 | 62 | 31 | 57 | 31 | 27 |
| Profile is manageable without treatment | 101 (48.6) | 23 (37.1) | 11 (35.5) | 30 (52.6) | 17 (54.8) | 20 (74.1) |
| Patient request | 34 (16.4) | 8 (12.9) | 12 (38.7) | 5 (8.8) | 5 (16.1) | 4 (14.8) |
| Patient concerns over side effects | 33 (15.9) | 13 (21.0) | 2 (6.5) | 8 (14.0) | 7 (22.6) | 3 (11.1) |
| Disease-modifying treatment considered last option of care | 3 (1.4) | 0 (0.00) | 1 (3.2) | 1 (1.8) | 1 (3.2) | 0 (0.0) |
| Symptoms not severe enough to warrant treatment | 43 (20.7) | 17 (27.4) | 6 (19.4) | 10 (17.5) | 2 (6.5) | 8 (29.6) |
| Diagnosed too recently | 36 (17.3) | 12 (19.4) | 6 (19.4) | 9 (15.8) | 3 (9.7) | 6 (22.2) |
| Lack of information in current treatment guidelines | 10 (4.8) | 4 (6.5) | 0 (0.0) | 2 (3.5) | 4 (12.9) | 0 (0.0) |
| Lack of evidence of the efficacy of current drug treatments available | 9 (4.3) | 4 (6.5) | 1 (3.2) | 0 (0.0) | 4 (12.9) | 0 (0.0) |
| Due to the underlying autoimmune disease | 10 (4.8) | 1 (1.6) | 5 (16.1) | 1 (1.8) | 2 (6.5) | 1 (3.7) |
| No approved therapies specifically for ILD | 8 (3.9) | 4 (6.5) | 0 (0.0) | 1 (1.8) | 2 (6.5) | 1 (3.7) |
| Medication is not covered by the patient’s insurance | 2 (1.0) | 1 (1.6) | 0 (0.0) | 0 (0.0) | 0 (0.0) | 1 (3.7) |
| Waiting for new drugs / products to be approved by regulatory bodies | 7 (3.4) | 4 (6.5) | 0 (0.0) | 2 (3.5) | 1 (3.2) | 0 (0.0) |
| Other | 14 (6.7) | 5 (8.1) | 1 (3.2) | 2 (3.5) | 3 (9.7) | 3 (11.1) |

ILD, interstitial lung disease; UK, United Kingdom.

**Table S14. Patients currently receiving treatment and reasons for never prescribing treatment by physician specialty**

|  | **All patients** | **Physician specialty** | | |
| --- | --- | --- | --- | --- |
|  |  | **Pulmonologist** | **Rheumatologist** | **Internal medicine** |
| Patients currently prescribed a treatment for their ILD, n (%) | 1335 | 790 | 484 | 61 |
| Yes | 1039 (77.8) | 589 (74.56) | 400 (82.64) | 50 (81.97) |
| No, but previously prescribed treatment | 88 (6.6) | 69 (8.73) | 18 (3.72) | 1 (1.64) |
| No, has never been prescribed treatment | 208 (15.6) | 132 (16.71) | 132 (16.71) | 10 (16.39) |
| Reason for never prescribing treatment for ILD, n (%) | 208 | 132 | 66 | 10 |
| Profile is manageable without treatment | 101 (48.6) | 57 (43.18) | 38 (57.58) | 6 (60.00) |
| Patient request | 34 (16.4) | 26 (19.70) | 7 (10.61) | 1 (10.00) |
| Patient concerns over side effects | 33 (15.9) | 27 (20.45) | 5 (7.58) | 1 (10.00) |
| Disease-modifying treatment considered last option of care | 3 (1.4) | 2 (1.52) | 1 (1.52) | 0 (0.00) |
| Symptoms not severe enough to warrant treatment | 43 (20.7) | 27 (20.45) | 11 (16.67) | 5 (50.00) |
| Diagnosed too recently | 36 (17.3) | 24 (18.18) | 11 (16.67) | 1 (10.00) |
| Lack of information in current treatment guidelines | 10 (4.8) | 8 (6.06) | 2 (3.03) | 0 (0.00) |
| Lack of evidence of the efficacy of current drug treatments available | 9 (4.3) | 7 (5.30) | 2 (3.03) | 0 (0.00) |
| Due to the underlying autoimmune disease | 10 (4.8) | 4 (3.03) | 5 (7.58) | 1 (10.00) |
| No approved therapies specifically for ILD | 8 (3.9) | 6 (4.55) | 2 (3.03) | 0 (0.00) |
| Medication is not covered by the patient’s insurance | 2 (1.0) | 2 (1.52) | 0 (0.00) | 0 (0.00) |
| Waiting for new drugs / products to be approved by regulatory bodies | 7 (3.4) | 7 (5.30) | 0 (0.00) | 0 (0.00) |
| Other | 14 (6.7) | 12 (9.09) | 2 (3.03) | 0 (0.00) |

ILD, interstitial lung disease.

**Table S15. Patients currently receiving treatment and reasons for never prescribing treatment by type of ILD**

|  | **Type of ILD** | | | | | | |
| --- | --- | --- | --- | --- | --- | --- | --- |
|  | **iNSIP** | **fHP** | **uILD** | **SSc-ILD** | **RA-ILD** | **PM/ DM-ILD** | **SS-ILD** |
| Patients currently prescribed a treatment for their ILD, n (%) | 263 | 225 | 133 | 263 | 269 | 83 | 99 |
| Yes | 213 (81.0) | 168 (74.7) | 72 (54.1) | 217 (82.5) | 217 (80.7) | 73 (88.0) | 79 (79.8) |
| No, but previously prescribed treatment | 19 (7.2) | 24 (10.7) | 15 (11.3) | 9 (3.4) | 9 (3.4) | 4 (4.8) | 8 (8.1) |
| No, has never been prescribed treatment | 31 (11.8) | 33 (14.7) | 46 (34.6) | 37 (14.1) | 43 (16.0) | 6 (7.2) | 12 (12.1) |
| Reason for never prescribing treatment for ILD, n (%) | 31 | 33 | 46 | 37 | 43 | 6 | 12 |
| Profile is manageable without treatment | 13 (41.9) | 17 (51.5) | 16 (34.8) | 17 (46.0) | 28 (65.1) | 3 (50.0) | 7 (58.3) |
| Patient request | 7 (22.6) | 8 (24.2) | 7 (15.2) | 5 (13.5) | 4 (9.3) | 1 (16.7) | 2 (16.7) |
| Patient concerns over side effects | 7 (22.6) | 7 (21.2) | 11 (23.9) | 2 (5.4) | 4 (9.3) | 1 (16.7) | 1 (8.3) |
| Disease-modifying treatment considered last option of care | 1 (3.2) | 0 (0.0) | 1 (2.2) | 1 (2.7) | 0 (0.0) | 0 (0.0) | 0 (0.0) |
| Symptoms not severe enough to warrant treatment | 6 (19.4) | 8 (24.2) | 10 (21.7) | 9 (24.3) | 7 (16.3) | 3 (50.0) | 0 (0.0) |
| Diagnosed too recently | 10 (32.3) | 3 (9.1) | 8 (17.4) | 4 (10.8) | 7 (16.3) | 2 (33.3) | 2 (16.7) |
| Lack of information in current treatment guidelines | 0 (0.0) | 3 (9.1) | 4 (8.7) | 1 (2.7) | 1 (2.3) | 0 (0.0) | 1 (8.3) |
| Lack of evidence of the efficacy of current drug treatments available | 1 (3.2) | 3 (9.1) | 3 (6.5) | 1 (2.7) | 1 (2.3) | 0 (0.0) | 0 (0.0) |
| Due to the underlying autoimmune disease | 0 (0.0) | 0 (0.0) | 0 (0.0) | 5 (13.5) | 4 (9.3) | 0 (0.0) | 1 (8.3) |
| No approved therapies specifically for ILD | 1 (3.2) | 3 (9.1) | 0 (0.0) | 0 (0.0) | 1 (2.3) | 1 (16.7) | 2 (16.7) |
| Medication is not covered by the patient’s insurance | 1 (3.2) | 0 (0.0) | 1 (2.2) | 0 (0.0) | 0 (0.0) | 0 (0.0) | 0 (0.0) |
| Waiting for new drugs / products to be approved by regulatory bodies | 1 (3.2) | 1 (3.0) | 5 (10.9) | 0 (0.0) | 0 (0.0) | 0 (0.0) | 0 (0.0) |
| Other | 2 (6.5) | 3 (9.1) | 4 (8.7) | 3 (8.1) | 2 (4.7) | 0 (0.0) | 0 (0.0) |

fHP, fibrotic hypersensitivity pneumonitis; ILD, interstitial lung disease; iNSIP, idiopathic non-specific interstitial pneumonia; PM/DM-ILD, polymyositis- / dermatomyositis-associated ILD; RA‑ILD, rheumatoid arthritis-associated ILD; SSc-ILD, systemic sclerosis-associated ILD; SS‑ILD, Sjögren's-associated ILD; uILD, unclassifiable ILD.

**Table S16. Patient milestones by country**

|  | **All patients** | **Country** | | | | |
| --- | --- | --- | --- | --- | --- | --- |
|  |  | **France** | **Germany** | **Italy** | **Spain** | **UK** |
| Age at first symptom of ILD, years | 534 | 114 | 120 | 79 | 144 | 77 |
| Mean (SD) | 56.92 (12.01) | 57.24 (13.79) | 52.16 (9.51) | 56.98 (12.24) | 59.97 (11.20) | 58.14 (11.95) |
| Age at first HCP visit for ILD, years | 534 | 114 | 120 | 79 | 144 | 77 |
| Mean (SD) | 57.57 (12.12) | 58.00 (14.05) | 53.06 (9.83) | 57.97 (12.50) | 60.31 (11.12) | 58.47 (12.05) |
| Age at confirmed diagnosis of ILD, years | 534 | 114 | 120 | 79 | 144 | 77 |
| Mean (SD) | 58.22 (12.2) | 58.46 (14.08) | 53.58 (10.04) | 58.73 (12.55) | 61.18 (11.18) | 59.06 (11.95) |
| Duration from first symptoms to first HCP visit (months) | 534 | 114 | 120 | 79 | 144 | 77 |
| Mean (SD) | 7.81 (22.65) | 9.16 (16.10) | 10.79 (33.69) | 11.91 (24.01) | 4.08 (15.74) | 3.92 (17.33) |
| Duration between first HCP visit and confirmed diagnosis of ILD, months | 534 | 114 | 120 | 79 | 144 | 77 |
| Mean (SD) | 7.73 (12.83) | 5.52 (7.16) | 6.07 (13.33) | 9.03 (15.20) | 10.51 (14.46) | 7.08 (11.84) |

Data presented only for patients with data available for all milestones.

HCP, healthcare professional; ILD, interstitial lung disease; SD, standard deviation; UK, United Kingdom.

**Table S17. Patient milestones by physician specialty**

|  | **All patients** | **Physician specialty** | | |
| --- | --- | --- | --- | --- |
|  |  | **Pulmonologist** | **Rheumatologist** | **Internal medicine** |
| Age at first symptom of ILD, years | 534 | 335 | 169 | 30 |
| Mean (SD) | 56.92 (12.01) | 59.37 (11.46) | 54.56 (11.24) | 42.90 (10.09) |
| Age at first HCP visit for ILD, years | 534 | 335 | 169 | 30 |
| Mean (SD) | 57.57 (12.12) | 60.20 (11.54) | 54.92 (11.21) | 43.21 (10.22) |
| Age at confirmed diagnosis of ILD, years | 534 | 30 | 335 | 33 |
| Mean (SD) | 58.22 (12.2) | 43.63 (10.38) | 60.97 (11.58) | 56.74 (12.34) |
| Duration from first symptoms to first HCP visit (months) | 534 | 355 | 169 | 30 |
| Mean (SD) | 7.81 (22.65) | 9.93 (23.92) | 4.34 (21.49) | 3.67 (4.79) |
| Duration between first HCP visit and confirmed diagnosis of ILD, months | 534 | 335 | 169 | 30 |
| Mean (SD) | 7.73 (12.83) | 9.14 (13.95) | 5.44 (10.81) | 5.01 (6.62) |

Data presented only for patients with data available for all milestones.

HCP, healthcare professional; ILD, interstitial lung disease; SD, standard deviation.

**Table S18. Patient milestones by type of ILD**

|  | **All patients** | **Type of ILD** | | | | | | |
| --- | --- | --- | --- | --- | --- | --- | --- | --- |
|  |  | **iNSIP** | **fHP** | **uILD** | **SSc-ILD** | **RA-ILD** | **PM/DM-ILD** | **SS-ILD** |
| Age at first symptom of ILD, years | 534 | 116 | 109 | 47 | 101 | 102 | 26 | 33 |
| Mean (SD) | 56.92 (12.01) | 60.73 (10.89) | 58.52 (11.73) | 61.01 (12.35) | 52.10 (11.04) | 56.57 (10.96) | 47.63 (13.52) | 55.60 (12.59) |
| Age at first HCP visit for ILD, years | 534 | 116 | 109 | 47 | 101 | 102 | 26 | 33 |
| Mean (SD) | 57.57 (12.12) | 61.17 (10.93) | 59.58 (11.92) | 61.87 (12.49) | 52.55 (11.30) | 57.24 (10.79) | 47.85 (13.82) | 56.24 (12.34) |
| Age at confirmed diagnosis of ILD, years | 534 | 534 | 116 | 109 | 47 | 101 | 102 | 26 |
| Mean (SD) | 58.22 (12.2) | 58.22 (12.2) | 61.80 (11.05) | 60.39 (11.92) | 62.79 (12.39) | 53.05 (11.34) | 57.90 (10.84) | 48.23 (14.04) |
| Duration from first symptoms to first HCP visit (months) | 534 | 116 | 109 | 47 | 101 | 102 | 26 | 33 |
| Mean (SD) | 7.81  (22.65) | 5.32 (16.01) | 12.74 (27.04) | 10.34 (20.62) | 5.39 (20.47) | 7.95 (29.63) | 2.58 (11.24) | 7.76 (14.38) |
| Duration between first HCP visit and confirmed diagnosis of ILD, months | 534 | 116 | 109 | 47 | 101 | 102 | 26 | 33 |
| Mean (SD) | 7.73 (12.83) | 7.46 (9.67) | 9.60 (16.38) | 10.99 (11.95) | 5.84 (7.18) | 7.87 (17.77) | 4.50 (6.22) | 5.81 (7.40) |

Data presented only for patients with data available for all milestones.

fHP, fibrotic hypersensitivity pneumonitis; HCP, healthcare professional; ILD, interstitial lung disease; iNSIP, idiopathic non-specific interstitial pneumonia; PM/DM-ILD, polymyositis- / dermatomyositis-associated ILD; RA‑ILD, rheumatoid arthritis-associated ILD; SD, standard deviation; SSc-ILD, systemic sclerosis-associated ILD; SS‑ILD, Sjögren's-associated ILD; uILD, unclassifiable ILD.

**Table S19. Other conditions suspected or investigated before confirmed diagnosis of ILD by country**

|  | **All patients** | **Country** | | | | |
| --- | --- | --- | --- | --- | --- | --- |
|  |  | **France** | **Germany** | **Italy** | **Spain** | **UK** |
| Other conditions suspected / investigated before diagnosis of ILD confirmed, n (%) | 232 | 35 | 54 | 58 | 52 | 33 |
| Angina | 4 (1.72) | 1 (2.86) | 0 (0.00) | 0 (0.00) | 0 (0.00) | 3 (9.09) |
| Anxiety | 29 (12.50) | 4 (11.43) | 2 (3.70) | 13 (22.41) | 5 (9.62) | 5 (15.15) |
| Asthma | 52 (22.41) | 3 (8.57) | 6 (11.11) | 15 (25.86) | 17 (32.69) | 11 (33.33) |
| Acute bronchitis | 14 (6.03) | 2 (5.71) | 3 (5.56) | 4 (6.90) | 3 (5.77) | 2 (6.06) |
| Bronchiectasis | 5 (2.16) | 2 (5.71) | 0 (0.00) | 0 (0.00) | 2 (3.85) | 1 (3.03) |
| Bronchitis | 24 (10.34) | 2 (5.71) | 3 (5.56) | 12 (20.69) | 4 (7.69) | 3 (9.09) |
| COPD | 91 (39.22) | 11 (31.43) | 42 (77.78) | 16 (27.59) | 11 (21.15) | 11 (33.33) |
| Connective tissue disease | 9 (3.88) | 1 (2.86) | 1 (1.85) | 5 (8.62) | 2 (3.85) | 0 (0.00) |
| Congestive heart failure | 18 (7.76) | 6 (17.14) | 3 (5.56) | 2 (3.45) | 3 (5.77) | 4 (12.12) |
| Emphysema | 5 (2.16) | 0 (0.00) | 2 (3.70) | 0 (0.00) | 0 (0.00) | 3 (9.09) |
| Influenza | 2 (0.86) | 0 (0.00) | 0 (0.00) | 2 (3.45) | 0 (0.00) | 0 (0.00) |
| GERD | 20 (8.62) | 4 (11.43) | 0 (0.00) | 9 (15.52) | 3 (5.77) | 4 (12.12) |
| Heart failure | 18 (7.76) | 3 (8.57) | 8 (14.81) | 4 (6.90) | 3 (5.77) | 0 (0.00) |
| Lack of fitness | 12 (5.17) | 0 (0.00) | 6 (11.11) | 4 (6.90) | 0 (0.00) | 2 (6.06) |
| Lung cancer | 3 (1.29) | 0 (0.00) | 2 (3.70) | 1 (1.72) | 0 (0.00) | 0 (0.00) |
| Myocardial infarction | 4 (1.72) | 1 (2.86) | 1 (1.85) | 1 (1.72) | 0 (0.00) | 1 (3.03) |
| Obesity | 7 (3.02) | 0 (0.00) | 0 (0.00) | 4 (6.90) | 0 (0.00) | 3 (9.09) |
| Pneumonia | 12 (5.17) | 3 (8.57) | 3 (5.56) | 2 (3.45) | 3 (5.77) | 1 (3.03) |
| Pulmonary embolism | 1 (0.43) | 1 (2.86) | 0 (0.00) | 0 (0.00) | 0 (0.00) | 0 (0.00) |
| Pulmonary hypertension | 8 (3.45) | 0 (0.00) | 4 (7.41) | 2 (3.45) | 2 (3.85) | 0 (0.00) |
| Sarcoidosis | 2 (0.86) | 1 (2.86) | 0 (0.00) | 1 (1.72) | 0 (0.00) | 0 (0.00) |
| Tuberculosis | 2 (0.86) | 0 (0.00) | 0 (0.00) | 1 (1.72) | 1 (1.92) | 0 (0.00) |
| Other ILD | 2 (0.86) | 0 (0.00) | 0 (0.00) | 0 (0.00) | 2 (3.85) | 0 (0.00) |
| Other condition | 14 (6.03) | 4 (11.43) | 1 (1.85) | 3 (5.17) | 4 (7.69) | 2 (6.06) |
| None | 1 (0.43) | 1 (2.86) | 0 (0.00) | 0 (0.00) | 0 (0.00) | 0 (0.00) |
| Don't know | 0 (0.00) | 0 (0.00) | 0 (0.00) | 0 (0.00) | 0 (0.00) | 0 (0.00) |

COPD, chronic obstructive pulmonary disease; GERD, gastroesophageal reflux disease; ILD, interstitial lung disease; UK, United Kingdom.

**Table S20. Other conditions suspected or investigated before confirmed diagnosis of ILD by physician specialty**

|  | **All patients** | **Physician speciality** | | |
| --- | --- | --- | --- | --- |
|  |  | **Pulmonologist** | **Rheumatologist** | **Internal medicine** |
| Other conditions suspected / investigated before diagnosis of ILD confirmed, n (%) | 232 | 164 | 60 | 8 |
| Angina | 4 (1.72) | 4 (2.44) | 0 (0.00) | 0 (0.00) |
| Anxiety | 29 (12.50) | 16 (9.76) | 13 (21.67) | 0 (0.00) |
| Asthma | 52 (22.41) | 42 (25.61) | 10 (16.67) | 0 (0.00) |
| Acute bronchitis | 14 (6.03) | 9 (5.49) | 5 (8.33) | 0 (0.00) |
| Bronchiectasis | 5 (2.16) | 4 (2.44) | 1 (1.67) | 0 (0.00) |
| Bronchitis | 24 (10.34) | 12 (7.32) | 12 (20.00) | 0 (0.00) |
| COPD | 91 (39.22) | 60 (36.59) | 29 (48.33) | 2 (25.00) |
| Connective tissue disease | 9 (3.88) | 6 (3.66) | 2 (3.33) | 1 (12.50) |
| Congestive heart failure | 18 (7.76) | 12 (7.32) | 4 (6.67) | 2 (25.00) |
| Emphysema | 5 (2.16) | 3 (1.83) | 2 (3.33) | 0 (0.00) |
| Influenza | 2 (0.86) | 1 (0.61) | 1 (1.67) | 0 (0.00) |
| GERD | 20 (8.62) | 14 (8.54) | 6 (10.00) | 0 (0.00) |
| Heart failure | 18 (7.76) | 17 (10.37) | 1 (1.67) | 0 (0.00) |
| Lack of fitness | 12 (5.17) | 4 (2.44) | 8 (13.33) | 0 (0.00) |
| Lung cancer | 3 (1.29) | 2 (1.22) | 1 (1.67) | 0 (0.00) |
| Myocardial infarction | 4 (1.72) | 3 (1.83) | 1 (1.67) | 0 (0.00) |
| Obesity | 7 (3.02) | 4 (2.44) | 3 (5.00) | 0 (0.00) |
| Pneumonia | 12 (5.17) | 8 (4.88) | 2 (3.33) | 2 (25.00) |
| Pulmonary embolism | 1 (0.43) | 0 (0.00) | 0 (0.00) | 1 (12.50) |
| Pulmonary hypertension | 8 (3.45) | 7 (4.27) | 1 (1.67) | 0 (0.00) |
| Sarcoidosis | 2 (0.86) | 1 (0.61) | 0 (0.00) | 1 (12.50) |
| Tuberculosis | 2 (0.86) | 1 (0.61) | 1 (1.67) | 0 (0.00) |
| Other ILD | 2 (0.86) | 2 (1.22) | 0 (0.00) | 0 (0.00) |
| Other condition | 14 (6.03) | 9 (5.49) | 5 (8.33) | 0 (0.00) |
| None | 1 (0.43) | 0 (0.00) | 0 (0.00) | 1 (12.50) |
| Don't know | 0 (0.00) | 0 (0.00) | 0 (0.00) | 0 (0.00) |

COPD, chronic obstructive pulmonary disease; GERD, gastroesophageal reflux disease; ILD, interstitial lung disease.

**Table S21. Other conditions suspected or investigated before confirmed diagnosis of ILD by type of ILD**

|  | **All patients** | **Type of ILD** | | | | | | |
| --- | --- | --- | --- | --- | --- | --- | --- | --- |
|  |  | **iNSIP** | **fHP** | **uILD** | **SSc-ILD** | **RA-ILD** | **PM/DM-ILD** | **SS-ILD** |
| Other conditions suspected / investigated before diagnosis of ILD confirmed, n (%) | 232 | 58 | 55 | 31 | 31 | 33 | 9 | 15 |
| Angina | 4 (1.72) | 2 (3.45) | 0 (0.00) | 2 (6.45) | 0 (0.00) | 0 (0.00) | 0 (0.00) | 0 (0.00) |
| Anxiety | 29 (12.50) | 7 (12.07) | 3 (5.45) | 2 (6.45) | 8 (25.81) | 3 (9.09) | 2 (22.22) | 4 (26.67) |
| Asthma | 52 (22.41) | 10 (17.24) | 24 (43.64) | 7 (22.58) | 3 (9.68) | 5 (15.15) | 0 (0.00) | 3 (20.00) |
| Acute bronchitis | 14 (6.03) | 4 (6.90) | 3 (5.45) | 2 (6.45) | 3 (9.68) | 2 (6.06) | 0 (0.00) | 0 (0.00) |
| Bronchiectasis | 5 (2.16) | 1 (1.72) | 1 (1.82) | 1 (3.23) | 2 (6.45) | 0 (0.00) | 0 (0.00) | 0 (0.00) |
| Bronchitis | 24 (10.34) | 4 (6.90) | 5 (9.09) | 3 (9.68) | 4 (12.90) | 3 (9.09) | 1 (11.11) | 4 (26.67) |
| COPD | 91 (39.22) | 23 (39.66) | 20 (36.36) | 10 (32.26) | 8 (25.81) | 21 (63.64) | 3 (33.33) | 6 (40.00) |
| Connective tissue disease | 9 (3.88) | 2 (3.45) | 0 (0.00) | 2 (6.45) | 5 (16.13) | 0 (0.00) | 0 (0.00) | 0 (0.00) |
| Congestive heart failure | 18 (7.76) | 7 (12.07) | 3 (5.45) | 4 (12.90) | 0 (0.00) | 3 (9.09) | 0 (0.00) | 1 (6.67) |
| Emphysema | 5 (2.16) | 2 (3.45) | 1 (1.82) | 0 (0.00) | 0 (0.00) | 2 (6.06) | 0 (0.00) | 0 (0.00) |
| Influenza | 2 (0.86) | 0 (0.00) | 0 (0.00) | 0 (0.00) | 1 (3.23) | 0 (0.00) | 1 (11.11) | 0 (0.00) |
| GERD | 20 (8.62) | 2 (3.45) | 1 (1.82) | 6 (19.35) | 7 (22.58) | 3 (9.09) | 0 (0.00) | 1 (6.67) |
| Heart failure | 18 (7.76) | 9 (15.52) | 1 (1.82) | 6 (19.35) | 0 (0.00) | 1 (3.03) | 0 (0.00) | 1 (6.67) |
| Lack of fitness | 12 (5.17) | 2 (3.45) | 1 (1.82) | 0 (0.00) | 1 (3.23) | 5 (15.15) | 1 (11.11) | 2 (13.33) |
| Lung cancer | 3 (1.29) | 0 (0.00) | 1 (1.82) | 1 (3.23) | 0 (0.00) | 1 (3.03) | 0 (0.00) | 0 (0.00) |
| Myocardial infarction | 4 (1.72) | 0 (0.00) | 0 (0.00) | 3 (9.68) | 0 (0.00) | 1 (3.03) | 0 (0.00) | 0 (0.00) |
| Obesity | 7 (3.02) | 1 (1.72) | 0 (0.00) | 2 (6.45) | 2 (6.45) | 1 (3.03) | 0 (0.00) | 1 (6.67) |
| Pneumonia | 12 (5.17) | 2 (3.45) | 4 (7.27) | 0 (0.00) | 1 (3.23) | 2 (6.06) | 3 (33.33) | 0 (0.00) |
| Pulmonary embolism | 1 (0.43) | 0 (0.00) | 0 (0.00) | 0 (0.00) | 0 (0.00) | 0 (0.00) | 1 (11.11) | 0 (0.00) |
| Pulmonary hypertension | 8 (3.45) | 3 (5.17) | 1 (1.82) | 2 (6.45) | 1 (3.23) | 1 (3.03) | 0 (0.00) | 0 (0.00) |
| Sarcoidosis | 2 (0.86) | 0 (0.00) | 1 (1.82) | 0 (0.00) | 0 (0.00) | 0 (0.00) | 1 (11.11) | 0 (0.00) |
| Tuberculosis | 2 (0.86) | 1 (1.72) | 0 (0.00) | 0 (0.00) | 1 (3.23) | 0 (0.00) | 0 (0.00) | 0 (0.00) |
| Other ILD | 2 (0.86) | 0 (0.00) | 0 (0.00) | 1 (3.23) | 0 (0.00) | 0 (0.00) | 0 (0.00) | 1 (6.67) |
| Other condition | 14 (6.03) | 5 (8.62) | 2 (3.64) | 1 (3.23) | 2 (6.45) | 2 (6.06) | 1 (11.11) | 1 (6.67) |
| None | 1 (0.43) | 0 (0.00) | 0 (0.00) | 0 (0.00) | 0 (0.00) | 0 (0.00) | 0 (0.00) | 1 (6.67) |
| Don't know | 0 (0.00) | 0 (0.00) | 0 (0.00) | 0 (0.00) | 0 (0.00) | 0 (0.00) | 0 (0.00) | 0 (0.00) |

COPD, chronic obstructive pulmonary disease; fHP, fibrotic hypersensitivity pneumonitis; GERD, gastroesophageal reflux disease; HCP, healthcare professional; ILD, interstitial lung disease; iNSIP, idiopathic non-specific interstitial pneumonia; PM/DM-ILD, polymyositis- / dermatomyositis-associated ILD; RA‑ILD, rheumatoid arthritis-associated ILD; SSc-ILD, systemic sclerosis-associated ILD; SS‑ILD, Sjögren's-associated ILD; uILD, unclassifiable ILD.
